# YB-1 recruitment to stress granules in zebrafish cells reveals a differential adaptive response to stress

**DOI:** 10.1038/s41598-019-45468-6

**Published:** 2019-06-21

**Authors:** Andrea Maria Guarino, Giuseppe Di Mauro, Gennaro Ruggiero, Nathalie Geyer, Antonella Delicato, Nicholas S. Foulkes, Daniela Vallone, Viola Calabrò

**Affiliations:** 1 University of Naples Federico II, Department of Biology, Monte Sant’Angelo Campus, Via Cinthia 4, Naples, 80126 Italy; 2 Institute of Toxicology and Genetics, Karlsruhe Institute of Technology, Hermann-von-Helmholtz-Platz 1, 76344 Eggenstein-Leopoldshafen, Germany; 3 University of Ferrara, Department of Life Sciences and Biotechnology, Via Borsari 46, 44121 Ferrara, Italy

**Keywords:** Fluorescence imaging, Stress signalling

## Abstract

The survival of cells exposed to adverse environmental conditions entails various alterations in cellular function including major changes in the transcriptome as well as a radical reprogramming of protein translation. While in mammals this process has been extensively studied, stress responses in non-mammalian vertebrates remain poorly understood. One of the key cellular responses to many different types of stressors is the transient generation of structures called stress granules (SGs). These represent cytoplasmic foci where untranslated mRNAs are sorted or processed for re-initiation, degradation, or packaging into mRNPs. Here, using the evolutionarily conserved Y-box binding protein 1 (YB-1) and G3BP1 as markers, we have studied the formation of stress granules in zebrafish (*D. rerio*) in response to different environmental stressors. We show that following heat shock, zebrafish cells, like mammalian cells, form stress granules which contain both YB-1 and G3BP1 proteins. Moreover, zfYB-1 knockdown compromises cell viability, as well as recruitment of G3BP1 into SGs, under heat shock conditions highlighting the essential role played by YB-1 in SG assembly and cell survival. However, zebrafish PAC2 cells do not assemble YB-1-positive stress granules upon oxidative stress induced by arsenite, copper or hydrogen peroxide treatment. This contrasts with the situation in human cells where SG formation is robustly induced by exposure to oxidative stressors. Thus, our findings point to fundamental differences in the mechanisms whereby mammalian and zebrafish cells respond to oxidative stress.

## Introduction

Stress caused by environmental insults or disease can disrupt cellular, tissue and organ homeostasis. Proteostasis, ribostasis and the appropriate regulation of the transcriptome, are often compromised under stress conditions. Stress-induced damage may culminate in cell or even organismal death^1^. Eukaryotes respond to detrimental conditions by activating a set of conserved processes that aim to re-establish cellular homeostasis. This multifaceted response is critical for cell survival^1^. It is characterized by stress-dependent changes in the transcriptome and down-regulation of global translation^2^. At the same time, the production of molecular chaperones is enhanced to promote the refolding or degradation of damaged proteins^3,4^.

After exposure to distinct environmental insults, such as oxidative stress, hypothermia or extreme heat, eukaryotic cells relocate proteins and messenger RNA into transient, dynamic structures known as Processing Bodies (PBs) and Stress Granules (SGs). PBs are associated with mRNA decay and contain decapping enzymes and scaffolding proteins^5^. Stress granules, instead, directly respond to the protein synthesis status of cells, and contain mRNAs, small ribosomal subunits and factors such as G3BP1 and YB-1 as their core components^6^. In SGs, messengers are sequestered and regulated following stressful conditions. They possess mRNA in a repressed state that may subsequently re-initiate translation in response to specific signals^7^.

Stress granules were initially described in tomato cells exposed to heat shock but they are also observed in other plants, protozoa, yeast, *Caenorhabditis elegans*, *Drosophila*, and mammalian cells^8–10^. Thus, SG assembly appears to represent a highly conserved cellular strategy to minimize stress-related damage and promote cell survival. Beyond their fundamental role in the stress response, SGs have also been implicated in human pathology. Abnormalities in SG formation have been associated with cancer, neurodegeneration and viral infections^11^. In addition, SGs have been reported to promote oncogenesis by supporting cancer cell survival while defects in SG dynamics can accelerate neurodegeneration. Notably, proteins involved in the pathogenesis of Alzheimer’s disease, amyotrophic lateral sclerosis (ALS), frontotemporal dementia (FTD), spinocerebellar ataxia (SCA) and Huntington’s disease (FUS, hnRNPA1, SMN, TAU and TDP43) are also SG components^1^.

In addition to saving anabolic energy by preventing the synthesis of housekeeping proteins, SGs promote cell survival by sequestering pro-apoptotic factors and promoting the translation of stress activated messengers such as BCL2 and ATF4^12–14^. Furthermore, SGs are extremely dynamic structures changing shape, dimensions and protein content depending on the signaling pathways which have been activated and the types of stress stimuli experienced^6^. However, all SGs share mRNAs and RNA-binding proteins^15^. Although the composition of SG protein aggregates is well studied, their precise function remains unclear and how RNAs are sorted and regulated in SGs is still unknown^15–17^.

The YB-1 protein belongs to the highly conserved cold shock domain (CSD) protein family and plays a critical role in SG assembly^18^. CSDs are nucleic acid-binding modules with broad binding properties that are present in several prokaryotic and eukaryotic stress-inducible proteins. YB-1 has been implicated in several cellular processes including regulation of transcription and translation, pre-mRNA splicing, DNA repair and mRNA packaging^19^. Although YB-1 is mainly located in the cytoplasm, recent evidence has shown that YB-1 can shuttle between the cytoplasm and nucleus where it regulates gene expression and participates in DNA-damage repair^20^. Increasing evidence highlights the importance of YB-1 function in the oxidative stress response^18^. More specifically, in normal conditions YB-1 co-localizes with GW182 in Processing Bodies (PBs) while, during oxidative stress it interacts with G3BP1 and leads to SG formation as part of a pro-survival program^19^. YB-1 can also bind to tiRNAs (tRNA-derived stress-induced RNAs) and this interaction is required for packaging of tiRNA-repressed mRNAs into SGs.

Most of our current knowledge concerning the function and regulation of stress granules in vertebrates, as well as the contribution of regulatory factors such as YB-1, is based on studies of mammalian cells. However, very little is known about the regulation and function of stress granules formation in non-mammalian vertebrates. The zebrafish (*Danio rerio*) is a fresh-water fish of the cyprinid family, and it represents one of the most popular and versatile genetic models for environmental and human disease studies^21,22^. However, in zebrafish, only limited information concerning the presence and regulation of SG-like structures is available^23–25^. We have recently shown that the subcellular localization of YB-1 is regulated by the circadian clock in zebrafish^26^. Here, using zebrafish cells as a model system and YB-1 as a marker for SG assembly, we reveal that stress granule formation is encountered upon exposure to heat shock in a similar manner to mammalian cells. However, while oxidative stress readily induces SG formation in human cells, no SGs were detected in ROS treated zebrafish cells. These results are consistent with fundamental differences in the response of fish and mammalian cells to oxidative stress.

## Results

### YB-1 positive aggregates in PAC2 cells and fin tissue

**Figure 1:**
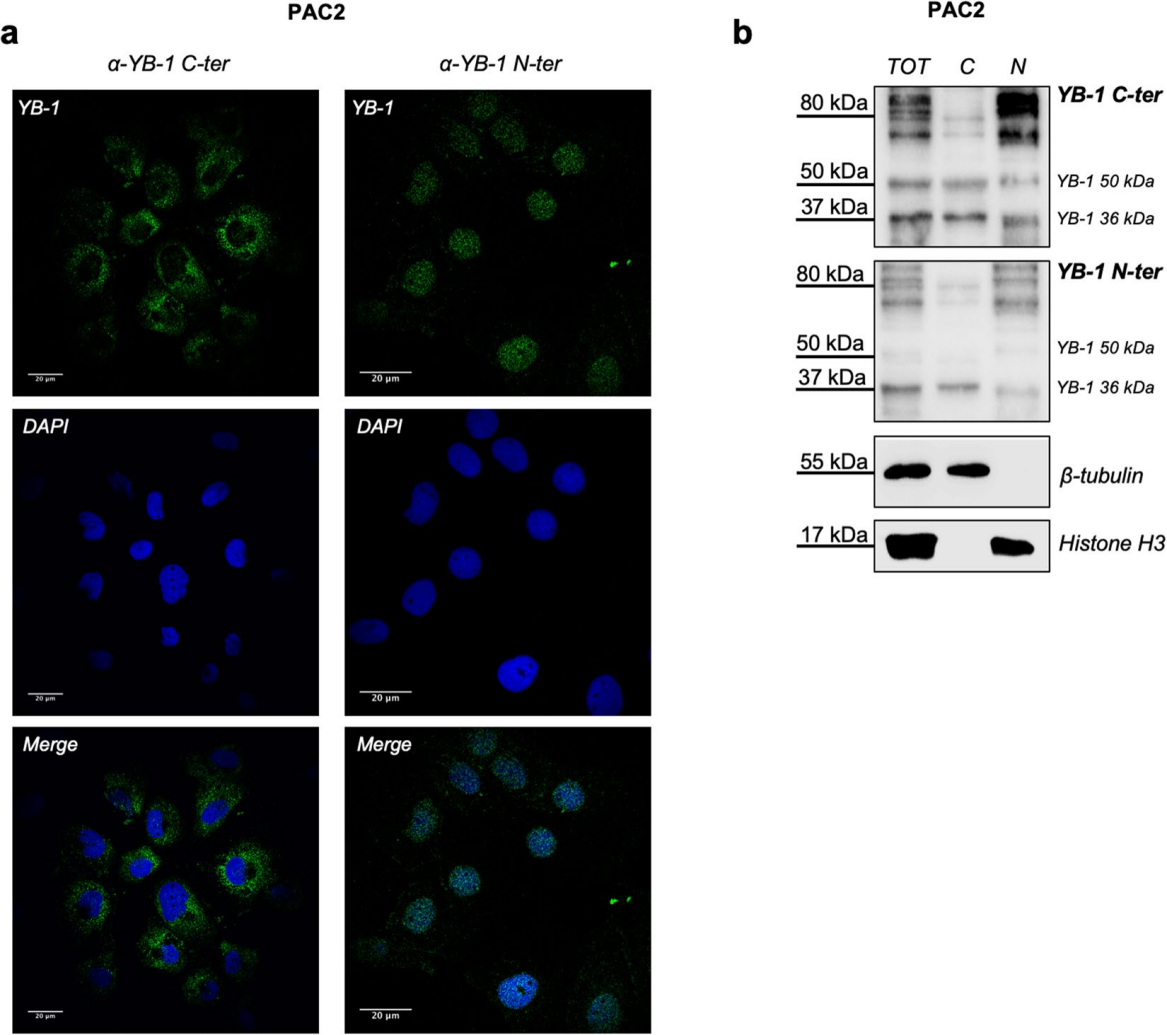
Immunoreactivity of human YB-1 antibodies against zebrafish YB-1. (**a)** confocal immunofluorescence analysis of PAC2 cells stained with human YB-1 antibodies (*α*-YB-1 C-ter and *α*-YB-1 N-ter), nuclei were stained with DAPI (blue); (**b**) western blot of PAC2 total (*TOT*), Cytoplasmic (**C**) and nuclear (N) protein extracts analysed with *α*-YB-1 N-ter and *α*-YB-1 C-ter antibodies; Histone H3 and ß-tubulin were used as loading controls for the nuclear and cytoplasmic extracts, respectively. Each panel is assembled from cropped western blotting images (see Supplementary Material file for the original images).

Many reports have documented that upon exposure to cellular stressors, the hYB-1 protein undergoes dynamic structural modifications leading to changes in its subcellular localization and function^27^. In order to use YB-1 to explore cytoplasmic stress granule formation in zebrafish cells under stress conditions, we exploited the high homology between human and zebrafish YB-1^26,28^ together with a set of antibodies that have been raised against specific portions of the human protein (Fig. S1). To assess which human YB-1 antibody was the more appropriate for visualizing cytoplasmic YB-1 protein, we performed immunofluorescence and western blot analysis of the zebrafish fibroblast-like PAC2 cell line using YB-1 antibodies raised against the C-terminal (C-ter YB-1) and N-terminal (N-ter YB-1) domains of the protein. As previously reported for mammalian cells^20^, immunofluorescence staining of the PAC2 cell line using the C-ter YB-1 antibody, detected a predominantly cytoplasmic signal which was distributed in a fine, punctate pattern (Fig. 1a, left panel). By western blot analysis, the same antibody recognized bands of about 50 and 36 kDa, previously described as the full length and truncated forms of the YB-1 protein, respectively^20,26,29^ which are localized in both nucleus and cytoplasm. It also recognized various high molecular weight, highly modified forms of YB-1^20,26,29^ (Fig. 1b) which appear to be predominantly nuclear proteins. On the contrary, by immunofluorescence assays performed in PAC2 cells, the N-ter YB-1 antibody detected mainly a nuclear signal and in western blot analysis it clearly detected all the forms recognized by the C-ter antibody with the exception of the 50 kDa band that was only barely detectable (Fig. 1a, right side of the panels and Fig. 1b). Based on these results and given the exclusively cytoplasmic localization described for SGs^1^, we chose to use the C-ter YB-1 antibody to visualize cytoplasmic YB-1-associated SG formation in response to different types of stress.

**Figure 2:**
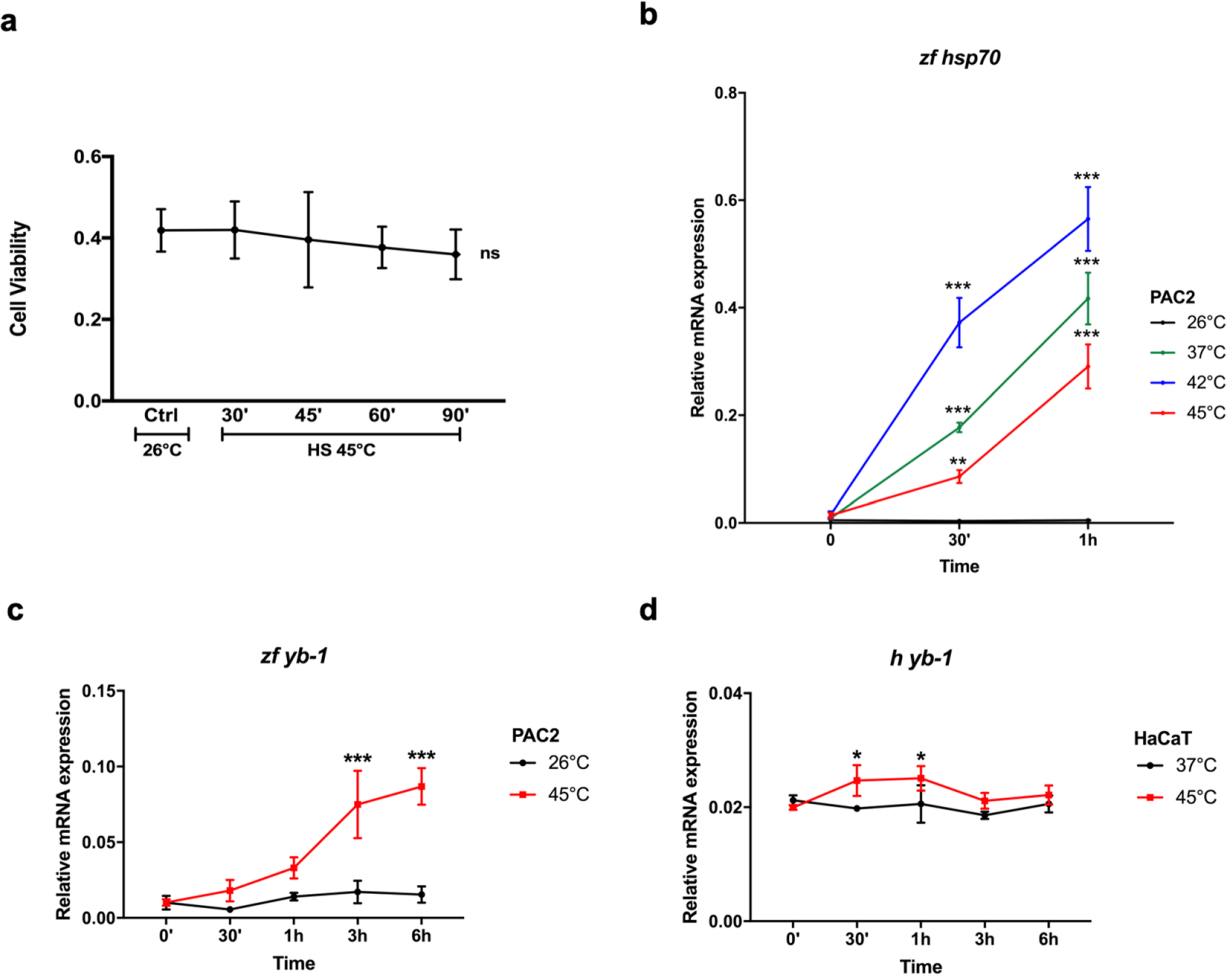
Heat shock induces *yb-1* transcript levels in zebrafish but not in mammalian cells. **(a)** cell viability of PAC2 cells after heat shock treatment at 45 °C. Cells were subjected to elevated temperatures for the times indicated on the x-axis and cell viability values (MTT) are plotted on the y-axis. Statistical analysis was performed using 1-way ANOVA followed by Dunnett’s multiple comparisons test; *ns* indicates no statistical significance (see also Table S1); (**b–d)** RT-qPCR analysis of zebrafish *zf hsp70* (**b**), *zf yb-1* (**c**) in PAC2 cells and human *h yb-1* (**d**) in HaCaT cells. Samples were taken at different time points during and after 1 hour of heat shock treatment at the indicated temperatures (for precise experimental details, see materials and methods section). Mean mRNA relative expression (n = 3) ± SD is plotted on the y-axes, whereas time is plotted on the x-axes. Statistical analysis was performed using 2-way ANOVA and Sidak’s multiple comparison test. Levels of significance between points of expression and time 0 are indicated (***p < 0.001, **p < 0.01, *p < 0.05) (see also Table S1 for statistical analysis).

In mammalian cells, YB-1 has been reported to localize in SGs upon heat shock treatment^18^. Therefore, we subjected the zebrafish cells to a heat shock by transferring them abruptly from 26 °C to 37 °C, 42 °C or 45 °C and then examined the impact on mRNA expression and the distribution of the zfYB-1 protein. Incubation at the highest temperature did not result in a significant reduction in PAC2 cell viability over a 90′ period (Fig. 2a), however, consistent with a robust heat shock response, expression of the heat shock gene *zf hsp70* was strongly induced by incubation of the cells at all three elevated temperatures (Fig. 2b). Furthermore, in PAC2 cells during 45 °C heat shock, *zf yb-1* mRNA levels were also strongly increased for the entire duration of the experiment (6 hours) (Fig. 2c) compared with a shallow, transient induction observed during the first hour following heat shock in the human HaCaT cell line (Fig. 2d). This data suggests that *yb-1* mRNA up-regulation may represent a species-specific aspect of the heat shock response.

**Figure 3:**
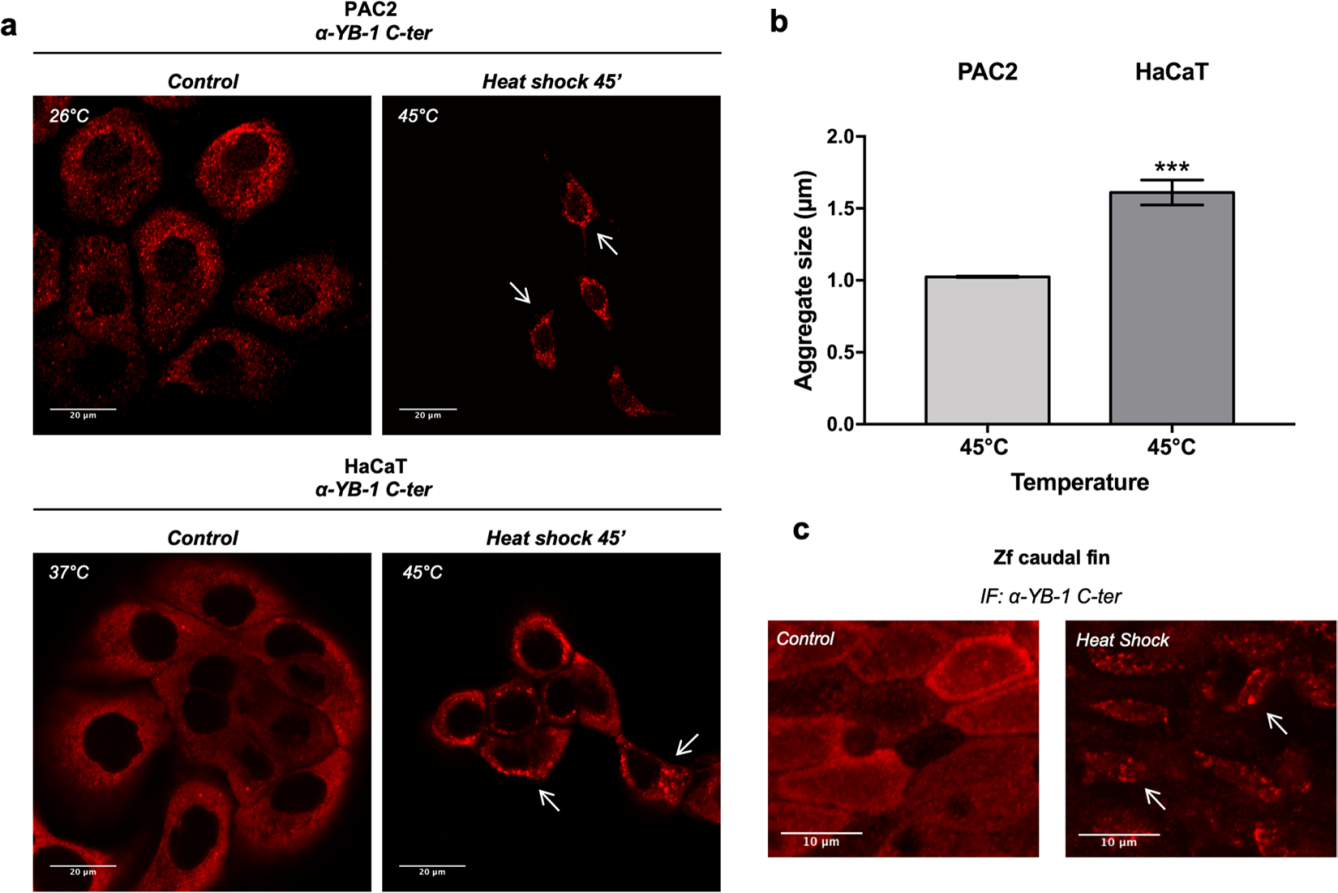
Heat shock promotes assembly of YB-1 positive aggregates in zebrafish and mammalian cells. **(a)** confocal immunofluorescence of PAC2 cells (*upper panels*) and HaCaT cells (*lower panels*) stained with human *α*-YB-1 C- ter antibody (red); some YB-1 aggregates are indicated by arrows; (**b)** quantification of YB-1 aggregate dimensions after heat shock at 45 °C for 45′ in PAC2 and HaCaT cells (unpaired t-test with Welch’s correction *p = 0.007, see also Table S1); (**c**) confocal immunofluorescence of adult zebrafish caudal fins at 26 °C (control) and after heat shock at 45 °C, stained with human *α*-YB-1 C-ter antibody. YB-1 aggregates are indicated by arrows.

**Figure 4:**
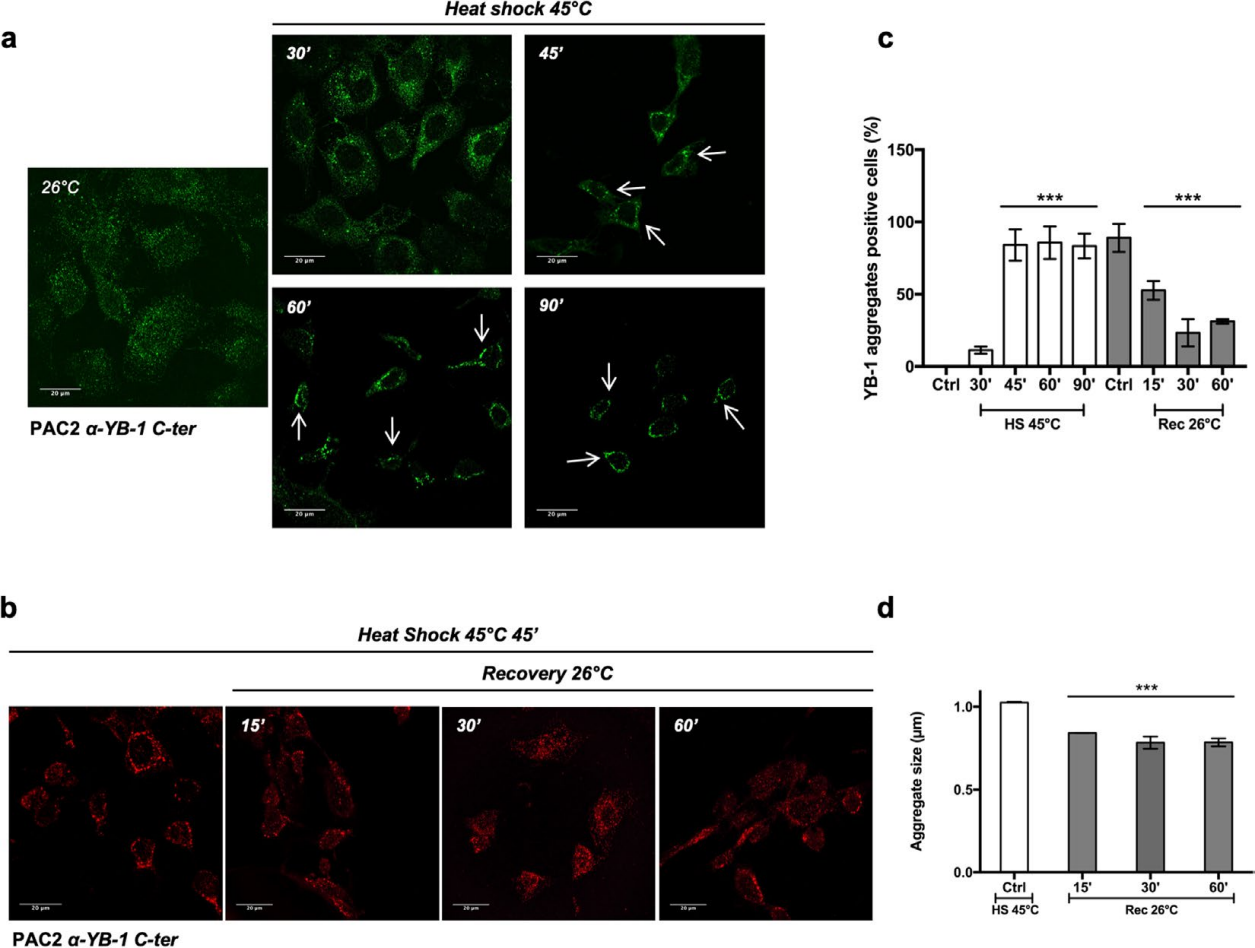
Kinetics of YB-1 aggregate formation. (**a**) confocal immunofluorescence of YB-1 (Green) in PAC2 cells at 26 °C (control) or after heat shock at 45 °C for the indicated times; (**b**) confocal immunofluorescence for YB-1(red) in PAC2 cells incubated at 45 °C for 45′ and then replaced at 26 °C for recovery from the heat shock at the indicated times; (**c**) percentage of cells forming YB-1 positive aggregates (plotted on the y-axes) incubated at 45 °C for the indicated times (white bars) and then allowed to recover for 15, 30 and 60 minutes at 26 °C (grey bars). Statistical analysis was performed using 1-way ANOVA and Dunnett’s multiple comparisons. Levels of significance are indicated (***p < 0.001) (see also Table S1); (**d)** quantification of YB-1 positive aggregate dimensions in PAC2 cells incubated at 45 °C for 45 minutes (white bar) and then allowed to recover at 26 °C for the indicated times (grey bars). Statistical analysis was performed using 1-way ANOVA and Dunnett’s multiple comparisons. Levels of significance are indicated (***p < 0.001) (see also Table S1).

We next explored whether heat shock treatment induced changes in the subcellular localization of YB-1, including the formation of aggregates in zebrafish cells. We performed an immunofluorescence assay for YB-1 in PAC2 cells which had been heat shock treated at different temperatures (37 °C, 40 °C, 42 °C and 45 °C). Interestingly, only zebrafish cells subjected to the treatment at 45 °C exhibited perinuclear aggregates similar to those observed in mammalian HaCaT cells, (Figs 3a and S2a,b). These YB-1 aggregates showed a significantly increased diameter compared with the diffuse punctate cytoplasmic YB-1 distribution in untreated cells (Fig. S2c). However, the YB-1 aggregates observed in PAC2 cells appeared smaller (67% +/− 1.5%) compared to those in HaCaT cells (Fig. 3b). The formation of similar perinuclear YB-1 aggregates was also observed in adult zebrafish caudal fins which had been first clipped from the animal, and immediately subjected to heat shock at 45 °C for 45 minutes prior to fixation of the tissue and the YB-1 immunofluorescence assay (Fig. 3c). To explore in more detail the heat shock-induced formation of YB-1 aggregates, we decided to examine the dynamics of aggregate formation. Thus, we exposed PAC2 cells to 45 °C for different periods of time from 30 to 90 minutes. Our immunofluorescence data showed that YB-1 positive aggregates already began to concentrate in the perinuclear compartment after 30 minutes of incubation and, after 45 minutes YB-1 aggregates were exclusively perinuclear (Fig. 4a,c). We then tested whether this YB-1 aggregate formation could be reversed by abruptly returning the cells to 26 °C after 45 minutes of heat shock treatment. We observed a significant decrease in the percentage of cells exhibiting YB-1 aggregates, as well as a reduction in aggregate size after only 15 minutes following return to the lower temperature. (Fig. 4b–d). Thus, comparing these observations with previous reports^30^, the YB-1 aggregates formed in zebrafish cells after heat shock treatment at 45 °C appear to have similar properties to the classical SGs observed in mammalian cells.

### Heat shock induced YB-1 aggregates represent *bona fide* SGs

To test whether the heat-shock induced YB-1 positive aggregates in PAC2 cells indeed represent SGs, we performed immunofluorescence co-localization experiments using antibodies against YB-1 and the stress granule assembly factor 1 (G3BP1). G3BP1 protein is a well know component of SGs in mammals and has been shown to initiate the assembly of SGs by forming a homo-multimeric and a hetero-multimeric complex with its close relative G3BP2^31^.

**Figure 5:**
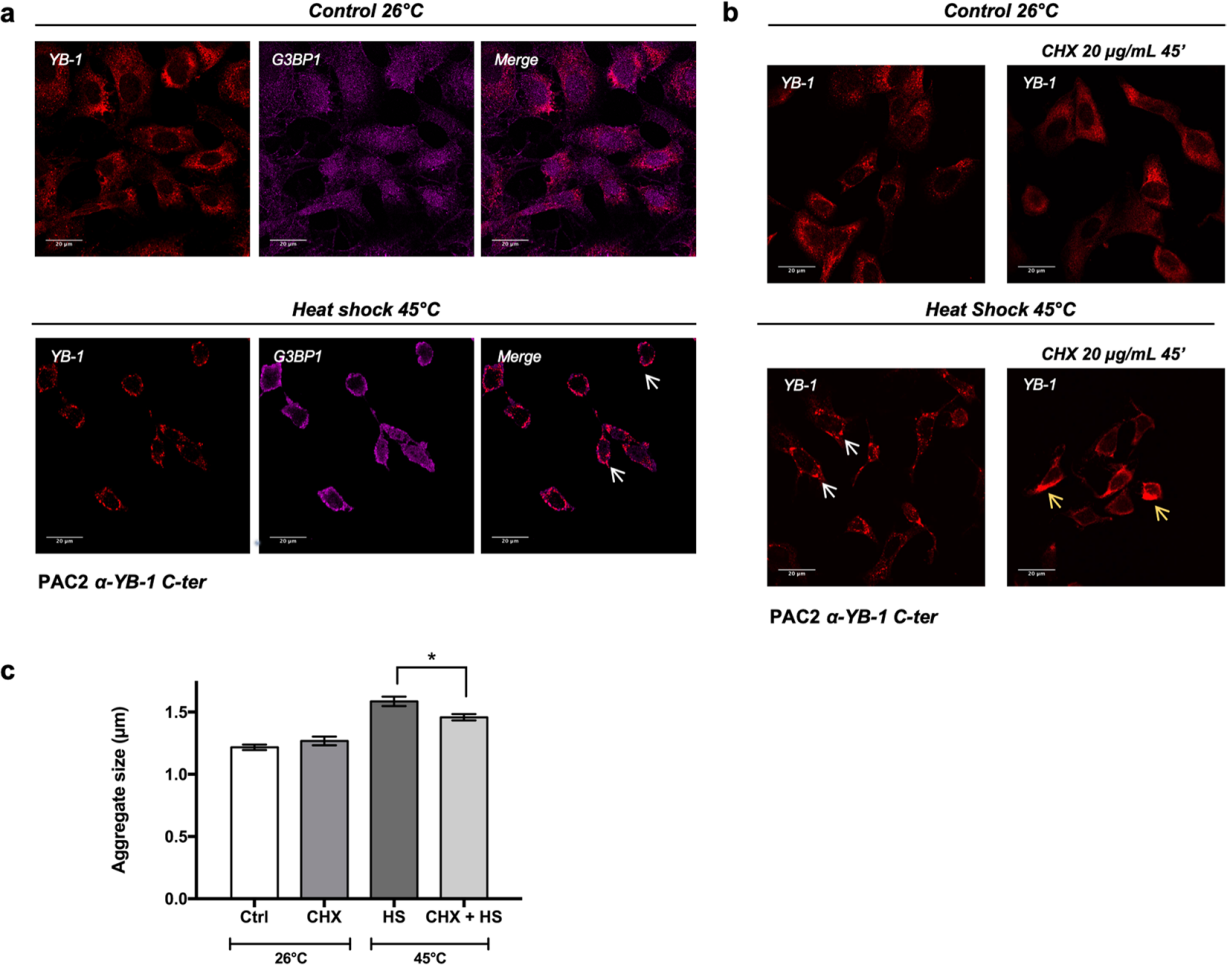
Heat shock induced YB-1 aggregates represent *bona fide* SGs. (**a**) Confocal immunofluorescence analysis of YB-1 (red) and G3BP1 (magenta) in PAC2 cells at 26 °C (control) or after heat shock at 45 °C for 45 minutes. Cellular colocalization is present in perinuclear aggregates indicated by white arrows; (**b**) confocal immunofluorescence of YB-1 in PAC2 cells treated with cycloheximide (20 µg/ml) at 26 °C (upper part of the panel) or after heat shock at 45 °C for 45 minutes (lower part of the panel). Controls are represented in the left part of the panel. White arrows indicate YB-1 positive aggregates (lower panel, left). Yellow arrows indicate the bulk of the protein not forming aggregates (lower panel, right); (**c**) quantification of YB-1 positive SG aggregate dimensions in PAC2 cells documented in the cycloheximide treatment analysis presented in panel b. Statistical analysis was performed using 1-way ANOVA followed by Tukey’s multiple comparisons test. Levels of significance are indicated (*p = 0.05) (see also Table S1).

We first verified cross-reactivity of human G3BP1 antibodies with the zebrafish ortholog by western blot and immunofluorescence assays in PAC2 cells (Fig. S3). In control zebrafish cells cultured at 26 °C, the signal from the anti G3BP1 antibody was almost uniformly distributed within the cytoplasmic compartment of the cells (Fig. 5a, upper panel and S3b). However, following the shift to 45 °C, we observed G3BP1 and YB-1 co-localization in perinuclear aggregates (Fig. 5a, lower panels). In mammalian cells, it has previously been demonstrated that cycloheximide prevents SG aggregation as a consequence of the blockade of protein synthesis^32,33^. Thus, to further confirm the SG identity of the YB-1 positive aggregates in zebrafish cells, we inhibited protein synthesis in PAC2 cells by treatment with cycloheximide prior to and during heat shock treatment. Consistent with the previous results in mammalian cells, in heat shock-treated zebrafish cells cycloheximide treatment significantly reduced (p < 0.05) the diameter of YB-1 positive aggregates (Fig. 5b,c), although YB-1 continued to show a diffuse perinuclear localization.

**Figure 6:**
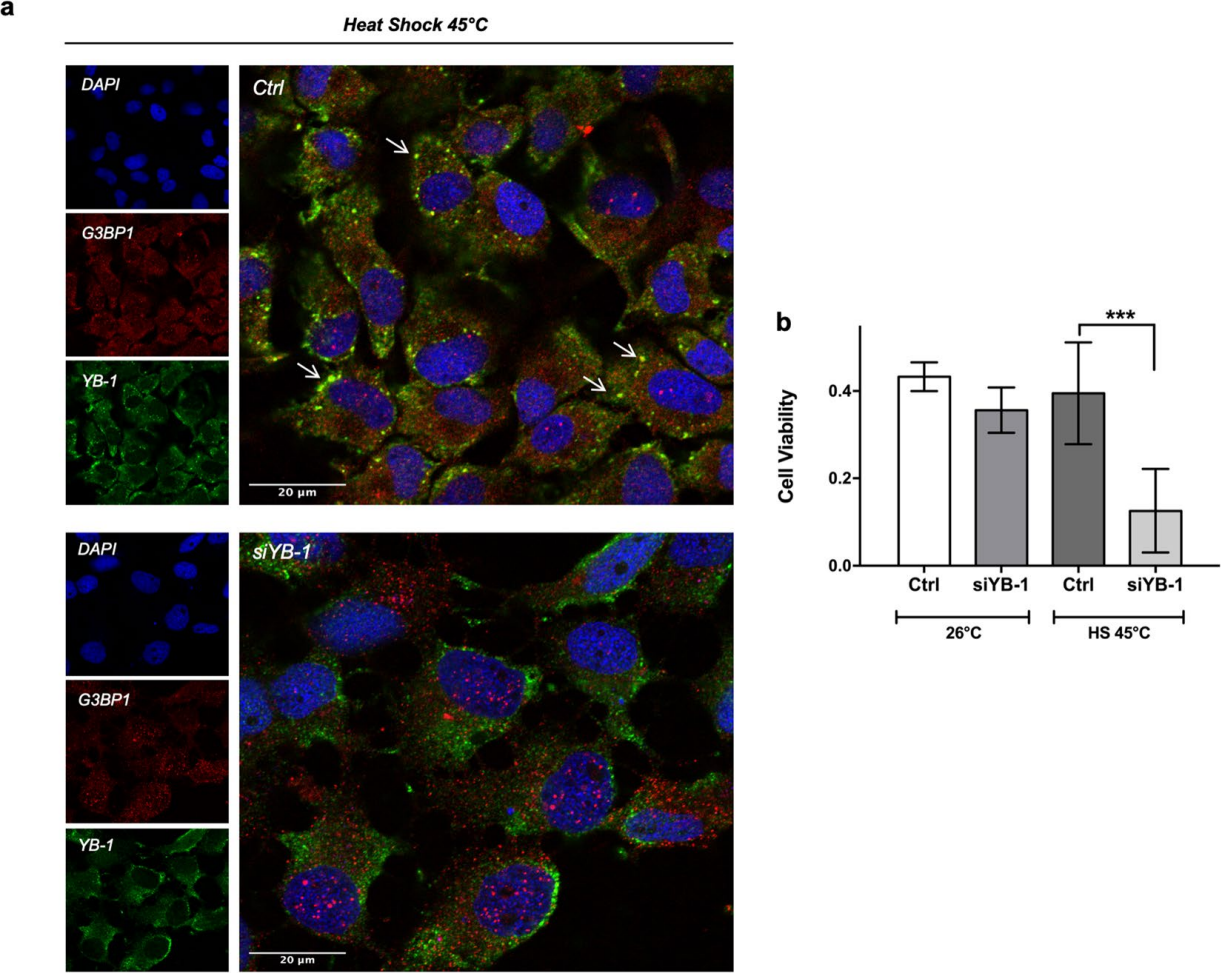
YB-1 is essential for SG formation and PAC2 cell viability upon thermal stress. (**a**) Confocal immunofluorescence analysis of G3BP1 (red) and YB-1 (green) in control (upper panel) and YB-1-silenced PAC2 cells (lower panel) after heat shock treatment (45 minutes at 45 °C). Nuclei were stained with DAPI (blue) and both single and merged images are presented. White arrows indicate SGs; (**b)** cell viability assay (MTT) in control or YB-1-silenced PAC2 cells at 26 °C or after heat shock (45 minutes at 45 °C). Statistical analysis was performed using 1-way ANOVA followed by Tukey’s multiple comparisons test. Levels of significance are indicated (***p 0.001) (see also Table S1).

Previous studies in mammalian cells have highlighted a key role for YB-1 within the context of SGs for cell survival upon stress conditions^18^. We therefore assessed the consequence of reducing YB-1 protein expression by siRNA silencing on the survival of heat shock treated PAC2 cells. We transfected PAC2 cells with a siYB-1 already successfully used in our previous studies^26^. This resulted in a reduction of all the immunoreactive YB-1 forms in western blot analysis, as well as a reduced immunofluorescent YB-1 signal (Fig. S4a,b, respectively). Strikingly, upon 45 minutes of heat shock treatment at 45 °C we failed to detect the typical pattern of perinuclear G3BP1/YB-1- positive SG aggregates in cells with reduced YB-1 expression (Fig. 6a). Moreover, we encountered a reduced cell viability in siYB-1- transfected cells compared with control cells that were maintained at 26 °C (Fig. 6b). Together, our data confirm that in zebrafish cells YB-1 perinuclear aggregates which form after heat shock treatment are *bona fide* SGs and point to an essential functional role played by YB-1 in SG assembly and cell survival under thermal stress.

### Differential response to ROS

**Figure 7:**
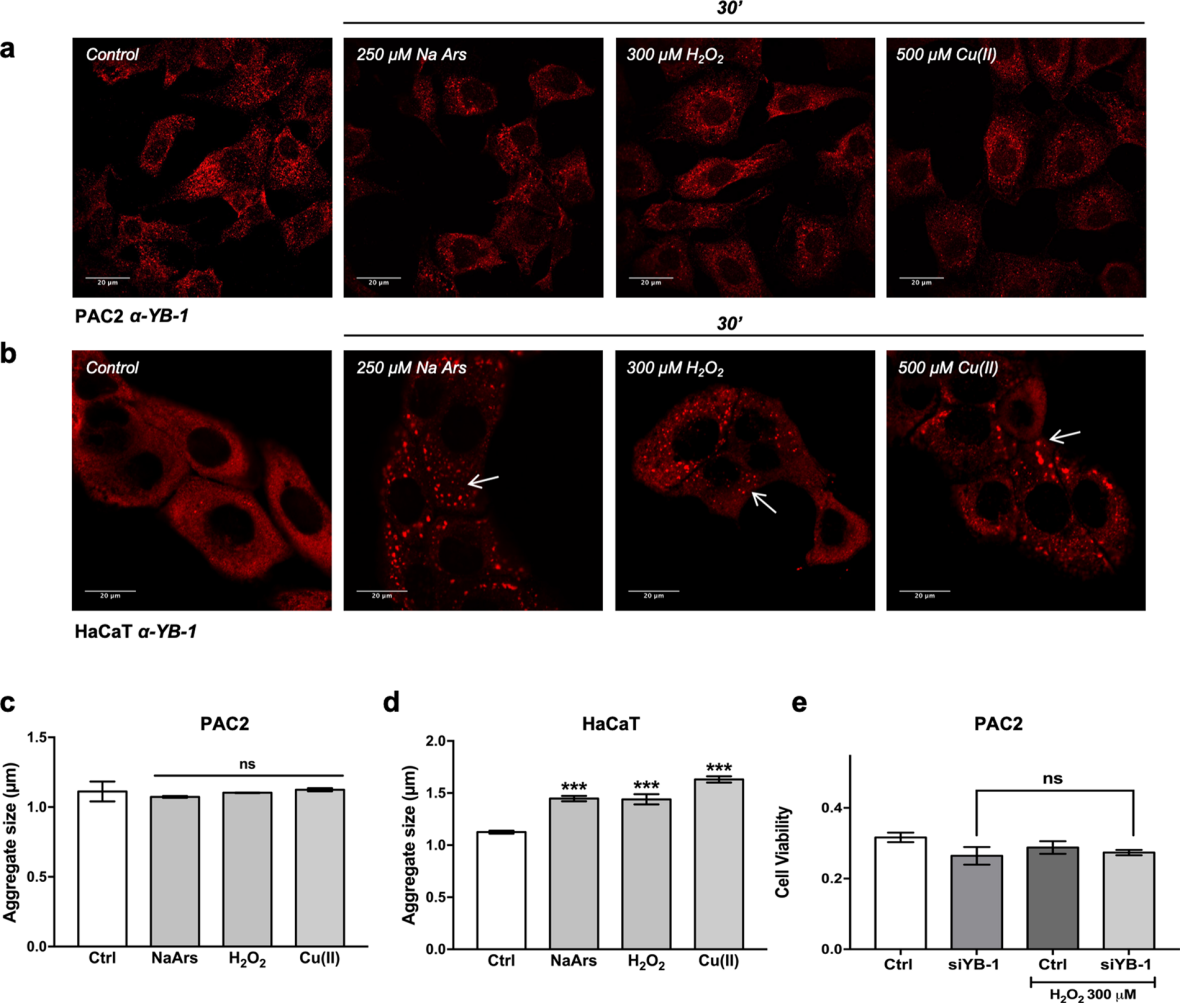
Oxidative stressors induce SGs in mammalian but not in zebrafish cells. (**a,b)** Representative confocal immunofluorescence for YB-1 (red) in (**a**) PAC2 cells and (**b**) HaCaT cells treated with 250 µM Na Ars, 300 µM H_2_O_2_ and 500 µM Cu(II) for 30 minutes. YB-1 positive SGs present in HaCaT cells are indicated by white arrows. (**c,d**) Quantification of aggregate size for the immunofluorescence analysis presented in panels a and b, respectively. (**e)** Cell viability assay (MTT) in control or YB-1-silenced PAC2 cells at 26 °C or after 300 μM H_2_O_2_ treatment for 45 minutes. Statistical analysis was performed using 1-way ANOVA followed by Tukey’s multiple comparisons test (see also Table S1).

Heat shock is not the only type of stress treatment that has been shown to induce the formation of stress granules in mammalian cells. Oxidative stress also represents one of the key environmental stressors triggering SG formation^1^. We have previously revealed major differences in the transcriptional response to ROS between mammalian and zebrafish cells^34,35^. We therefore questioned whether differences in the SG response to oxidative stress might also exist between mammalian and zebrafish cells. Thus, we incubated PAC2 cells for 30 minutes or 1 hour with three typical oxidative stressors at working concentrations according to those used in previous publications^18^: sodium arsenite (Na Ars, 250 μM), hydrogen peroxide (H_2_O_2_, 300 μM) and copper (Cu II, 500 μM). Interestingly, by immunofluorescence assay we failed to detect either significant changes in YB-1 cytoplasmic distribution or the formation of aggregate structures resembling SGs (Figs 7a,c and S5a). Conversely, similar treatments in mammalian HaCaT cells, efficiently induced YB-1 positive SGs (Fig. 7b,d). Furthermore, we did not observe a reduction of YB1-silenced PAC2 cells viability upon treatment with hydrogen peroxide, thus indicating that YB-1 does not play a pro-survival role under oxidative stress, at least under the experimental conditions tested (Fig. 7e).

To explore the possibility that SG-like aggregation in zebrafish cells may occur at higher doses of oxidative stressors, we exposed PAC2 cells to a broad range of concentrations of Na Ars, H_2_O_2_ and copper (from 600 μM to 1.5 mM). Although with higher concentrations we observed an enrichment of the perinuclear localization of YB-1and G3BP1, we failed to detect the formation of YB-1 positive SGs aggregates even at the highest concentrations of oxidative stressors (Fig. S6). Importantly, acute changes in the expression of genes which have previously been shown to be linked with the cellular response to oxidative stress (*jun-B*, *jun-D*, *c-fos*, *cry1a* and *cry5*)^34^ were already observed in PAC2 cells treated with 300 μM H_2_O_2_ (Fig. S5b).

**Figure 8:**
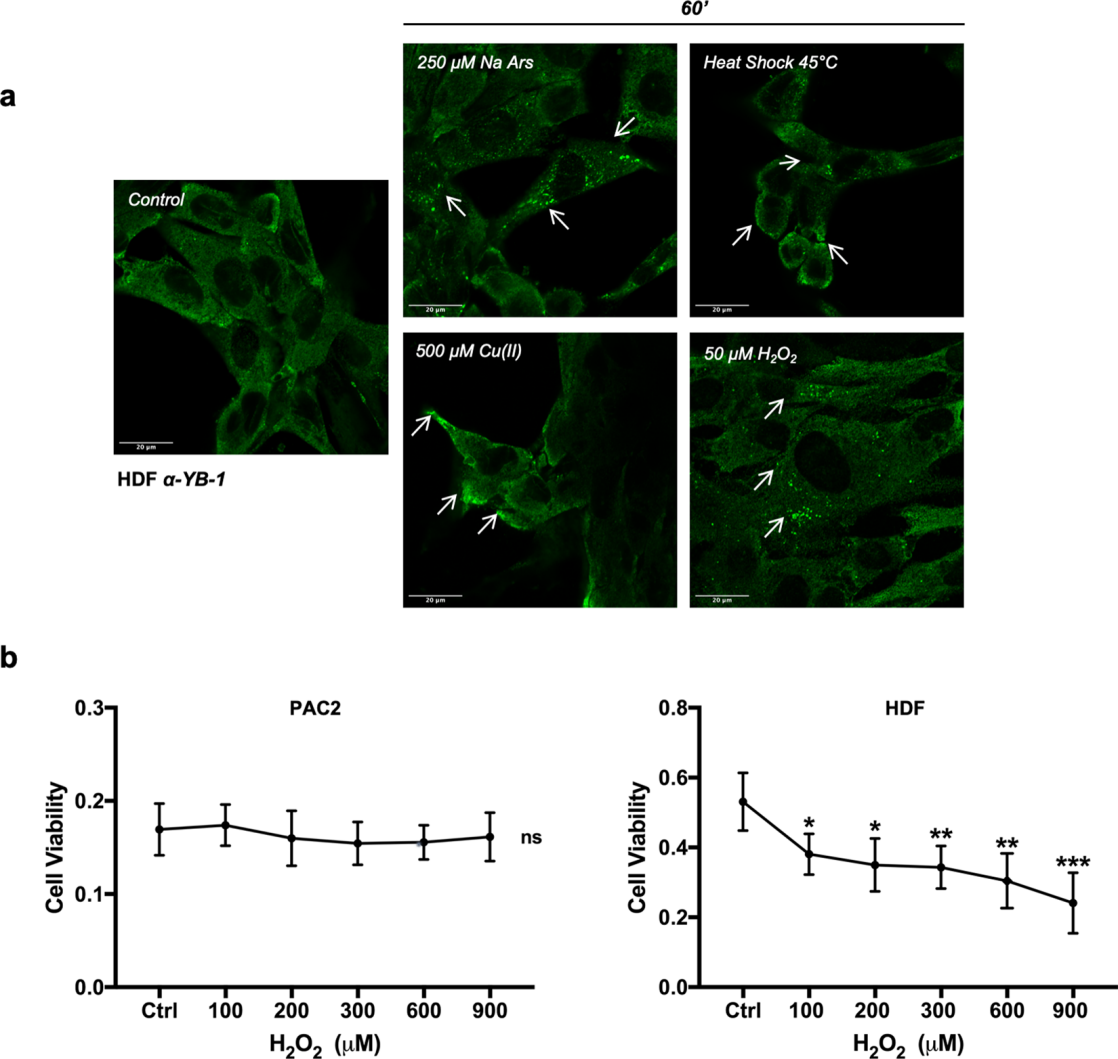
Heat shock, Arsenite, Copper and H_2_O_2_ treatment promotes assembly of YB-1 positive aggregates in human HDF fibroblast cells. (**a)** Representative confocal immunofluorescence images of HDF cells stained with human *α*-YB-1 C- ter antibody (green). Some YB-1 aggregates are indicated by arrows. (**b)** Cell viability assay (MTT) in PAC2 and HDF cells after 45 minutes treatment with different doses of H_2_O_2_. Statistical analysis was performed using 1-way ANOVA followed by Tukey’s multiple comparisons test. Levels of significance are indicated (***p 0.001) (see also Table S1).

We next wished to exclude that this differential response to stressors observed in PAC2 cells compared with mammalian cells may be simply due to the different cell type origin of these two cell lines (fibroblast-like for PAC2 cells and keratinocyte for HaCaT cells). We performed an immunofluorescence assay for YB-1 in a human dermal fibroblast cell line (HDF) after heat shock at 45 °C, as well as upon Na Ars (250 μM), H_2_O_2_ (50 μM) and Cu II (500 μM) treatments. As for the HaCaT cell line, and consistent with previous reports for many other human cell lines^36–38^, all the stressors used were able to induce SG formation in HDF cells (Fig. 8a). Interestingly, we also observed a reduction of cell viability in human fibroblasts after treatment with H_2_O_2_ whereas zebrafish PAC2 fibroblasts were almost unaffected by the same treatment (Fig. 8b), thus indicating a lower sensitivity of zebrafish cells to oxidative stress.

## Discussion

Stress granule formation has been observed in plants, protozoa, yeast, *C. elegans*, *Drosophila* and mammalian cells. Despite enormous interest in SGs due to their possible contribution to the pathogenesis of several human diseases, many aspects of SG function are poorly understood. In particular, little is known about how SG formation is tailored to the particular environmental challenges faced by different species. Here, we have revealed that in zebrafish fibroblast cells, heat shock induces the formation of SGs in a comparable manner to that described in mammalian cells. In contrast, while ROS treatment robustly induces SG formation in mammalian cells, there is no SG response in PAC2 zebrafish cells.

Our study has exploited YB-1 protein as a marker for stress granule formation in zebrafish. However, our understanding of the precise role played by YB-1 in SG assembly remains incomplete. Previous studies highlighted the role of YB-1 in formation of stress granules^18^, whereas in other reports, YB-1 silencing apparently does not alter the formation of SGs^39,40^. In our study, consistent with Somasekharan, S. P. *et al*.^18^, we reveal that knock down of YB-1 expression in zebrafish cells leads to impaired SG assembly and reduced cell viability following heat shock. In human cells, YB-1 protein was first reported to indirectly increase SG formation during oxidative stress by translationally activating G3BP1, a nucleator for SG assembly^18^. YB-1 was then shown to bind to tiRNA via its cold shock domain to package tiRNA-repressed mRNAs into SGs, a pathway that is independent of G3BP1^41^. Previous studies have revealed that YB-1 function is predominantly regulated by posttranslational modifications, protein cleavage and subcellular compartmentalization^26,27,42^. Indeed, YB-1 is abundant and constitutively expressed in multiple human tissues and its expression is further induced in tumor cells or following DNA damage by E box binding transcription factors such as c-Myc^43^ and Twist^44^. Interestingly, we have revealed that YB-1 mRNA expression is induced upon heat shock in zebrafish but not in mammalian cells suggesting that in fish cells *de novo* YB-1 protein synthesis is an essential requirement for the pro-survival response to thermal stress. It is tempting to speculate that mammalian cells, being more sensitive to thermal stress, are more reliant on a pre-existing pool of YB-1 protein to allow an immediate response following heat shock.

### Conservation of Stress Granule formation in response to heat shock

Our results revealing that heat shock induces stress granule formation in both zebrafish and mammalian cells are consistent with this function representing a highly conserved facet of the cellular response to heat shock. In both cell types these granules are a site of colocalization of YB-1 and G3BP1 and exhibit a perinuclear distribution. Furthermore, similar kinetics was observed for the assembly of YB-1 positive SGs at 45 °C in mammalian and zebrafish cells. However, while in mammalian cells an increase of 8 °C (37 °C to 45 °C) is sufficient to trigger SGs formation, for zebrafish cells a temperature shift of 19 °C (26 °C to 45 °C) is required. Furthermore, after 45 minutes incubation at 45 °C, more than 90% of zebrafish cells were still viable and able to recover from stress upon return to 26 °C. This contrasts with the situation in mammalian cells where a moderate temperature elevation (37 °C to 45 °C) for a relatively short time (30–45 minutes) can reduce cell survival to 10%^45^. These data indicate that zebrafish cells are generally more resistant to temperature changes than mammalian cells, only mounting a SG response after a relatively large increase in temperature and also showing lower mortality under elevated temperatures. It is tempting to speculate that this reflects the fact that zebrafish is an ectothermic organism living in shallow, slow-flowing water, and so may have adapted to frequent changes in body temperature^46^. Indeed, zebrafish as ectotherms, show a greater tolerance of environment-induced changes in body temperature^47^ compared with endothermic mammals.

### Species- and stressor-specific differences in SG assembly

We have revealed significant differences in SG formation in response to heat shock and oxidative stress between zebrafish and mammalian cells. Our own data and that of previous reports fail to indicate any cell type-specificity in mammalian stress granule formation in response to stress. However, consistent with the general notion of an inherent plasticity in SG function, recent data have pointed to stress-specific differences in the composition and dynamics of SGs in mammalian cells^48^. For example, accumulation of heat shock proteins (HSPs) including HSP27 and HSP70 is the most prominent part of the complex cellular response to hyperthermic conditions in all type of organisms. HSP27 is only found in SGs induced by heat shock, but is absent in sodium arsenite induced SGs^49^. Furthermore, while mRNAs encoding house-keeping proteins are recruited to heat-shock induced SGs, transcripts encoding HSP70 proteins are selectively excluded^50^. HSP90 mRNA transcripts, instead, are selectively excluded from oxidative-stress induced SGs^51^. Stress and species-specific differences have also been reported in the mechanisms regulating SG formation in mammalian cells. For example, mammalian cells use two key regulatory mechanisms to rapidly shut down general protein synthesis. The first involves eIF2α phosphorylation by stress-activated eIF2a kinases (eIF2αK)^52,53^, while a second mechanism involves p-4E-BPs dephosphorylation that prevents the assembly of eIF4F and inhibits translation initiation^54^. eIF2α phosphorylation is required for SG assembly in mammalian cells, but not in *Drosophila*, *C. elegans* or yeast. Furthermore, in mammalian cells, certain types of stress, including oxidative stress, are strictly reliant on eIF2Fa phosphorylation for the promotion of SG formation while heat shock also induces SGs through 4E-BP activation^55^. A HAP1 human cell line expressing a non-phosphorylatable form of eIF2α (S51A) was shown to be unable to assemble stress granules in response to sodium arsenite and was hypersensitive to the toxic effects of low doses of sodium arsenite. However, after heat shock, this cell line demonstrated no difference in its ability to induce SGs thus suggesting that heat shock may also function through alternative p-eIF2a-independent pathways that rely on the activation of 4E-BPs^48^.

We have shown that zebrafish fibroblasts exposed to oxidative stressors such as sodium arsenite, hydrogen peroxide and copper do not produce SGs, however, without any significant reduction of cell viability. Due to the limited repertoire of established, stable zebrafish cell lines available, we cannot exclude cell type-specificity in the stress response for this fish species. However, our data obtained from fibroblast cells points to the existence of alternative pathways, not involving SG assembly that may operate in zebrafish cells helping them to tolerate oxidative stress insults. In this regard, our previous data have already revealed significant differences in the transcriptional response to ROS between mammals and zebrafish cells. Indeed, while in mammalian cells, ROS-induced gene expression is mediated by various signaling proteins and transcription factors including NF-kB, PI3K, MAPK and p53, in zebrafish, the D-box enhancer appears to represent the principle oxidative stress responsive enhancer element^34^. This D-box mediated response to ROS in fish cells has been associated with the regulation of gene expression by direct exposure to visible light, a property which is absent in mammalian cells. Thus, together, our results point to fundamental changes in the mechanisms whereby vertebrate cells respond to oxidative stress during evolution.

## Methods

### Fish care, treatment and ethical statements

All husbandry and experimental procedures were performed in accordance with European Legislation for the Protection of Animals used for Scientific Purposes (Directive 2010/63/EU), the German Animal Protection Law (May 18^th^, 2006 (BGBl. I S. 1206, 1313), last changed March 29^th^, 2017 (BGBl. I S. 626). Research was also approved by the Local Government of Baden-Württemberg, Karlsruhe, Germany (35-9185.81/G-131/16). General license for fish maintenance and breeding: Az.: 35-9185.64.

Zebrafish (*Dario rerio*, Tübingen strain) were maintained according to standard procedures^56^ in a re-circulating water system at 26 °C and under 14:10 light: dark cycles. For our experiment 6–12 months old zebrafish males (n = 5) were used and chosen based on a health check. The caudal fins were amputated using razor blades following anesthesia with 0.02% w/v MS222 (3- aminobenzoate methanesulfonic acid, Sigma-Aldrich, St Louis, MO). Fish were left to recover in an isolated cage in the presence of 0.00005% methylene blue for 24 hours to avoid distress and for the health to be monitored.

### Cell culture

The zebrafish PAC2^57^ cell line was propagated at 26 °C in an atmospheric CO_2_, non-humidified cell culture incubator; cells were cultured in L-15 (Leibovitz) medium (Gibco BRL) supplemented with 15% Fetal Bovine Serum (Sigma-Aldrich, St Louis, MO), 100 units/ml penicillin, 100 µg/ml streptomycin and 50 µg/ml gentamicin (Gibco BRL).

HaCaT (human spontaneously immortalized keratinocytes from adult skin) and HDF (human primary dermal fibroblast) were purchased from Cell Line Service (CLS, Germany) and cultured in a humidified incubator at 37 °C and 5% CO_2_ in DMEM High Glucose (Gibco BRL) supplemented with 10% Fetal Bovine Serum (Gibco BRL), 1% L-Glutamine (Gibco BRL) and 1% Pen-Strep solution (Gibco BRL).

Cells were routinely checked for mycoplasma contamination, using a mycoplasma detection kit (abm, Canada).

### Immunofluorescence microscopy of zebrafish cells and zebrafish adult caudal fins

PAC2 cells were seeded on coverslips (6.0 × 10^4^ cells) and maintained in constant darkness for the subsequent 48 hours prior to treatment. HaCaT cells were seeded on coverslips (3.0 × 10^4^ cells) and treated after 24 hours. Following treatment, both mammalian and zebrafish cells were gently rinsed with 1X PBS and then fixed in PFA 3.7% for 10 and 15′, respectively. After 3 washes with 1X PBS (10 minutes each), cells were washed twice with 0.01% Tween-PBS and blocked for 1 hour with a 1% BSA-0.01% Tween-PBS solution for PAC2 and with a 3% BSA solution for mammalian cells to avoid non-specific binding of antibodies. PAC2 cells were then incubated overnight at 4 °C with the primary antibodies. Primary antibody incubation for mammalian cells was performed for 1 hour in darkness at room temperature. After incubation with primary antibodies, cells were washed three times with 0.01% Tween-PBS (10 minutes each) and incubated with secondary antibodies for 45 minutes in darkness. Then, both PAC2 and HaCaT cells were incubated for 5 minutes in a DAPI solution (1:50000) (Sigma-Aldrich, St Louis, MO) followed by 3 washes in 0.01% Tween PBS. Coverslips were immersed in a Dako mounting medium (Agilent).

Immunofluorescence analysis of zebrafish caudal fins was performed as previously reported^58^. Specifically, after an overnight fixation of the fin-clips in Carnoy’s solution (60% ethanol, 30% chloroform, 10% acetic acid) at 4 °C, fins were incubated overnight in 100% Methanol and then subjected to sequential rehydration steps of 10′ in 100%, 66% and 33% methanol in PBTX (1XPBS, 0,3% Triton X100). At this stage, fins were pre-incubated in PBTX plus 1% BSA blocking solution for 3 hours. Then the primary antibody was added and the samples were incubated at 4 °C overnight. After several washes, samples were incubated at 4 °C with the secondary fluorescent antibody overnight. DAPI staining was used for visualization of nuclei. Then samples were mounted on glass slides. All images were acquired using a Leica SPE confocal microscope (63x oil immersion objective) or Carl Zeiss LSM 700 (63x oil immersion objective) microscope. The size of aggregate/stress granules in confocal immunofluorescence images was measured with Fiji (ImageJ) software to calculate the Feret’s statistical diameter (Fig. S7). Feret’s statistical diameter is the perpendicular distance between parallel tangents touching opposite sides of the profile of elliptical/circular shaped particles. This parameter is a reliable indicator of aggregate shape and dimensions^59,60^. Analysis were performed on threshold images. Particles with areas outside the range of 0.2–2.0 µm and with size circularity values outside 1.00 were ignored.

### Cell viability assay

Cell viability was determined by the MTT 3-(4,5-dimethylthiazol-2-yl)-2,5-diphenyl tetrazolium bromide assay (Sigma-Aldrich, St Louis, MO). PAC2 cells were seeded in 96-well plates at 2.0 × 10^4^ per well and the assay was performed accordingly to the manufacturer´s instructions. The optical absorbance was determined at 570 nm using an iMark microplate reader (Bio-Rad, USA).

### siRNAs and transfections

PAC2 cells were transfected using Fugene HD (Promega) according to the manufacturer’s recommendations. A 4:1 ratio (4 µl of Fugene HD reagent for each µg of siRNA) was used. Briefly, cells were seeded at 70–80% confluence (1.5 × 10^6^) in 100-mm dishes; after 48 hours of incubation in darkness, cells were transiently transfected with siRNA (100 nM final concentration). 24 hours after siRNA transfection, cells were treated or left untreated. Negative Control siRNA, provided by Riboxx (Germany) as a pool of 3 different siRNAs, was used as a negative control. A predesigned siRNA for YB-1 was purchased from Riboxx (Germany):

zfYB-1 siRNA guide sequence (5′-3′): UUCUCCUUAUCCUCCUCUCCCCC zfYB-1 siRNA passenger sequence (5′-3′): GGGGGAGAGGAGGAUAAGGAGAA;

siRNA Negative Control-1 guide sequence (5′-3′): UUGUACUACACAAAAGUACCCCC siRNA Negative Control-1 passenger sequence (5′-3′): GGGGGUACUUUUGUGUAGUACAA

siRNA Negative Control-2 guide sequence (5′-3′): GAACGAAUUUAUAAGUGGCCCCC siRNA Negative Control-2 passenger sequence (5′-3′): GGGGGCCACUUAUAAAUUCGUUC

siRNA Negative Control-3 guide sequence (5′-3′): UUGUACUACACAAAAGUACCCCC siRNA Negative Control-3 passenger sequence (5′-3′): GGGGGUACUUUUGUGUAGUACAA.

### Antibodies

Primary antibodies: anti-YB-1 raised against amino acids 1 to 100 of hYB-1 protein (Abcam 12148 N-ter); anti-YB-1 raised against amino acids 307–324 of hYB-1 (Sigma-Aldrich, St Louis, MO, Y0396 C-ter); anti-vinculin (Sigma-Aldrich, St Louis, MO, V9131); anti-β-tubulin (H-235 Santa Cruz Biotechnology); anti-Histone H3 (9715 Cell Signaling); anti-G3BP1 (611127 BD transduction Laboratories, BD Biosciences).

Secondary fluorescent antibodies: Alexa Fluor 488 anti-rabbit (Thermo-Fisher Scientific); Alexa Fluor 647 anti-goat (Thermo-Fisher Scientific); Cy3 anti-rabbit (Jackson ImmunoResearch); DAPI (Sigma-Aldrich, St Louis, MO, D9542).

### Immunoblot analysis

For total protein extraction, 1.5 × 10^6^ cells were seeded in individual 100-mm petri dishes. After 48 hours, cells were harvested in Lysis Buffer (50 mM Tris-HCl pH 7.5, 5 mM EDTA, 150 mM NaCl, 1% NP-40, 0.5% sodium deoxycholate) with the addition of 1 mM phenylmethylsulfonyl fluoride and protease inhibitor cocktail (Sigma-Aldrich, St Louis, MO). Cells were detatched with a scraper and left on ice for 30′. Then extracts were clarified by centrifugation at 13200 rpm for 30′ at 4 °C.

For nuclear-cytoplasmic fractionation, 1.5 × 10^6^ cells were seeded in 100-mm dishes. 24 hours after seeding, cell lysates were fractionated to obtain cytoplasmic and nuclear fractions as previously reported^24^. The amount of protein in the samples was determined by the Bio-Rad protein assay (Bio-Rad, Milan, Italy).

After the addition of Laemmli buffer (Sigma-Aldrich, St Louis, MO) samples were boiled at 100 °C for 5 min and resolved by SDS- polyacrylamide gel electrophoresis (SDS-PAGE). About 10 μg of nuclear and 30 μg of cytoplasmic or total extracts (1:3 rate) were separated by SDS-PAGE.

Proteins were then transferred to a polyvinylidene difluoride membrane (PDVF, Millipore) using a Mini trans-blot apparatus (Bio-Rad, Milan, Italy) according to the manufacturer’s instructions. The PVDF membrane was blocked in 5% w/v milk buffer (5% w/v non-fat dried milk, 50 mM Tris, 200 mM NaCl, 0.2% Tween 20) and incubated overnight at 4 °C with primary antibodies diluted in 5% w/v milk or bovine serum albumin (BSA) buffer according to the manufacturer’s instructions. Following three washes with TTBS (Tris-buffered saline, 0.1% Tween), the blots were incubated for 1 hour at RT with HRP-conjugated secondary antibodies (Sigma-Aldrich, St Louis, MO). Proteins were visualized by an enhanced chemiluminescence method (ECL, Bio-Rad, Milan, Italy) and analyzed by Quantity One W software of ChemiDoc TM XRS system (Bio-Rad, Milan, Italy).

### Heat Shock treatments in cells and adult zebrafish caudal fins

Cells cultured in petri dishes were abruptly placed on the surface of a pre-heated water bath at the indicated time points and temperatures by floating the Petri dishes on a water bath floater (Promega).

Following heat shock treatment, cells were processed for immunofluorescence analysis. For the heat shock recovery experiment, cells were returned to a 26 °C incubator for the designated time.

For the analysis of mRNA expression during heat shock treatment, cells were exposed for 1 hour to heat shock and then returned to a 26 °C incubator for the remainder of the time course in order to avoid mortality due to prolonged exposure to the higher temperature.

Fins were gently placed in 2 ml Eppendorf tubes filled with complete cell culture medium (Leibovitz’s L-15, Gibco BRL) and placed in a pre-warmed water bath at 45 °C for the designated time or in a cell culture incubator at 26 °C as a control.

### Quantitative RT-PCRs

Total RNA was extracted with Trizol Reagent (Gibco) according to the manufacturer’s instructions. Reverse transcription was performed using Superscript III RT (Invitrogen). A StepOnePlus Real-Time qRT-PCR System (Applied Biosystems) and SYBR Green I fluorescent dye (Promega) were used. Expression levels were normalized using *zf β-actin* and *gapdh* mRNA expression for zebrafish PAC2 and human HaCaT cells, respectively. The relative levels of mRNA were calculated using the 2^−ΔΔCT^ method. For each gene, primer sequences are presented in Supplementary Table S2.

### Statistical analysis

Statistical analyses were performed using GraphPad Prism7 (GraphPad Software Inc). Statistical significance of difference in measured variables between control and treated groups was determined by t-test or analysis of variance (1-way or 2-way ANOVA) followed by Tukey’s, Dunnett’s, or Sidak’s multiple comparisons post-test depending on the experiment as specified in the figure legends and Table S1. To report p-values the NEJM (New England Journal of Medicine) decimal format was used; differences were considered statistically significant at *P < 0.033, **P < 0.002 and ***P < 0.001. Detailed statistical information is summarized in Table S1.

## Supplementary information

Supplementary Figures and Tables

Original Western-blot data
