# Supplementary material for: YB-1 recruitment to stress granules in zebrafish cells reveals a differential adaptive response to stress
Source: Sci Rep. 2019 Jun 21;9:9059. doi: 10.1038/s41598-019-45468-6 (PMC6588705; doi:10.1038/s41598-019-45468-6)
Supplement: Supplementary file 1 — Supplementary Figures and Tables [file 41598_2019_45468_MOESM1_ESM.docx]

**YB-1 recruitment to stress granules in zebrafish cells reveals a differential adaptive response to stress**

Andrea Maria Guarino^1^, Giuseppe Di Mauro^2,3^, Gennaro Ruggiero^2^, Nathalie Geyer^2^, Antonella Delicato^1^, Nicholas S. Foulkes^2^, Daniela Vallone^2*^ and Viola Calabrò^1*^.

^1^University of Naples Federico II, Department of Biology, Monte Sant’Angelo Campus, Via Cinthia 4, Naples, 80126, Italy.

^2^ Institute of Toxicology and Genetics, Karlsruhe Institute of Technology, Hermann-von-Helmholtz-Platz 1, 76344, Eggenstein-Leopoldshafen, Germany.

^3^ University of Ferrara, Department of Life Sciences and Biotechnology, Via Borsari 46, 44121, Ferrara, Italy.

*Co-senior and co-corresponding authors

Viola Calabrò, phone +39 081 679069 email: [vcalabro@unina.it](mailto:vcalabro@unina.it)

Daniela Vallone, phone +49 721 60828728 email: daniela.vallone@kit.edu

Key words: Y-box binding protein 1, cold shock domain, oxidative stress, heat shock, Danio rerio.

**Supplementary materials**

**Table S1: Statistical analysis**

| **Figure/experiment** | **Test method** | **Significance** | **n** |
| --- | --- | --- | --- |
| **Figure 2a** – Cell viability | Ordinary 1-way ANOVA | F = 0.7438; P = 0.57 ns | 6 |
|  | 95% confidence interval |  |  |
| Ctrl 26°C vs. 45°C 30' | Dunnett's multiple comparison test | P >0.99 ns |  |
| Ctrl 26°C vs. 45°C 45' | - | P = 0.95 ns |  |
| Ctrl 26°C vs. 45°C 60' | - | P = 0.73 ns |  |
| Ctrl 26°C vs. 45°C 90' | - | P = 0.46 ns |  |
| **Figure 2b** – RT-qPCR (*zf hsp70*) | 2-way ANOVA |  | 3 |
|  | 95% confidence interval |  |  |
|  | Interaction | F = 54.61; P <0.001*** |  |
|  | Time | F = 345.2; P <0.001*** |  |
|  | Temperature °C | F = 186; P <0.001*** |  |
| 0' - 26°C vs. 37°C | Dunnett's multiple comparison test | P >0.99 ns |  |
| 0' - 26°C vs. 42°C | - | P = 0.96 ns |  |
| 0' - 26°C vs. 45°C | - | P = 0.96 ns |  |
| 30' - 26°C vs. 37°C | - | P <0.001*** |  |
| 30' - 26°C vs. 42°C | - | P <0.001*** |  |
| 30' - 26°C vs. 45°C | - | P = 0.005** |  |
| 1h' - 26°C vs. 37°C | - | P <0.001*** |  |
| 1h' - 26°C vs. 42°C | - | P <0.001*** |  |
| 1h' - 26°C vs. 45°C | - | P <0.001*** |  |
| **Figure 2c** – RT-qPCR (*zf yb-1*) | 2-way ANOVA |  | 3 |
|  | 95% confidence interval |  |  |
|  | Interaction | F = 16.54; P <0.001*** |  |
|  | Time | F = 25.61; P <0.001*** |  |
|  | Temperature °C | F = 90.44; P <0.001*** |  |
| 26°C vs. 45°C | Sidak's multiple comparison test |  |  |
| 0' | - | P >0.99 ns |  |
| 30' | - | P = 0.45 ns |  |
| 1h | - | P = 0.1 ns |  |
| 3h | - | P <0.001*** |  |
| 6h | - | P <0.001*** |  |
| **Figure 2d** – RT-qPCR (*h yb-1*) | 2-way ANOVA |  | 3 |
|  | 95% confidence interval |  |  |
|  | Interaction | F = 2.995; P <0.04* |  |
|  | Time | F = 2.792; P <0.05 ns |  |
|  | Temperature °C | F = 14.53; P <0.001** |  |
| 37°C vs. 45°C | Sidak's multiple comparison test |  |  |
| 0' | - | P = 0.92 ns |  |
| 30' | - | P = 0.01* |  |
| 1h | - | P = 0.03* |  |
| 3h | - | P = 0.38 ns |  |
| 6h | - | P = 0.82 ns |  |
| **Figure 3b** – Aggregate size (µm) | Unpaired t-test (two-tailed) | P = 0.007** | 3 |
|  | with Welch's correction |  |  |
|  | 95% confidence interval |  |  |
| **Figure 4c** – *left part (white bars)* | Ordinary 1-way ANOVA | F = 87.27; P <0.001*** | 3 |
|  | 95% confidence interval |  |  |
| Ctrl 26°C vs 45°C 30' | Dunnett's multiple comparison test | P = 0.30 ns |  |
| Ctrl 26°C vs 45°C 45' | - | P <0.001*** |  |
| Ctrl 26°C vs 45°C 60' | - | P <0.001*** |  |
| Ctrl 26°C vs 45°C 90' | - | P <0.001*** |  |
| **Figure 4c** – *right part (black bars)* | Ordinary 1-way ANOVA | F = 45.77; P <0.001*** | 3 |
|  | 95% confidence interval |  |  |
| Ctrl 45°C 45' vs Rec 26°C 15' | Dunnett's multiple comparison test | P <0.001*** |  |
| Ctrl 45°C 45' vs Rec 26°C 30' | - | P <0.001*** |  |
| Ctrl 45°C 45' vs Rec 26°C 60' | - | P <0.001*** |  |
| **Figure 4d** – Aggregate size (µm) | Ordinary 1-way ANOVA | F = 157.7; P <0.001*** | 3 |
|  | 95% confidence interval |  |  |
| Ctrl 45°C 45' vs Rec 26°C 15' | Dunnett's multiple comparison test | P <0.001*** |  |
| Ctrl 45°C 45' vs Rec 26°C 30' | - | P <0.001*** |  |
| Ctrl 45°C 45' vs Rec 26°C 60' | - | P <0.001*** |  |
| **Figure 5c** – Aggregate size (µm) | Ordinary 1-way ANOVA | F = 60.78; P <0.001*** | 3 |
|  | 95% confidence interval |  |  |
| Ctrl vs. CHX | Tukey's multiple comparison test | P = 0.46 ns |  |
| Ctrl vs. HS 45°C 45' | - | P <0.001*** |  |
| Ctrl vs. CHX + HS | - | P = 0.005** |  |
| CHX vs. HS 45°C 45' | - | P = 0.002** |  |
| CHX 20 µg/mL vs. CHX + HS | - | P = 0.01* |  |
| HS 45°C 45' vs. CHX + HS | - | P = 0.05* |  |
| **Figure 6c** – Cell viability | Ordinary 1-way ANOVA | F = 17.19; P <0.001*** | 3 |
|  | 95% confidence interval |  |  |
|  | Tukey's multiple comparison test |  |  |
| Ctrl 26°C vs. siYB-1 26°C | - | P = 0.38 ns |  |
| Ctrl 26°C vs. Ctrl 45°C | - | P = 0.85 ns |  |
| Ctrl 26°C vs. siYB-1 45°C | - | P <0.001*** |  |
| siYB-1 26°C vs. Ctrl 45°C | - | P = 0.84 ns |  |
| siYB-1 26°C vs. siYB-1 45°C | - | P <0.001*** |  |
| Ctrl 45°C vs. siYB-1 45°C | - | P <0.001*** |  |
| **Figure 7c** – Aggregate size (µm) | Ordinary 1-way ANOVA | F = 1.093; P = 0.41 ns | 3 |
| *PAC2* | 95% confidence interval |  |  |
|  | Dunnett's multiple comparison test |  |  |
| Ctrl vs. NaArs | - | P = 0.45 ns |  |
| Ctrl vs. hydro. per. | - | P = 0.98 ns |  |
| Ctrl vs. Cu(II) | - | P = 0.95 ns |  |
| **Figure 7d** – Aggregate size (µm) | Ordinary 1-way ANOVA | F = 124.5; P <0.001*** | 3 |
| *HaCaT* | 95% confidence interval |  |  |
|  | Dunnett's multiple comparison test |  |  |
| Ctrl vs. NaArs | - | P <0.001*** |  |
| Ctrl vs. hydro. per. | - | P <0.001*** |  |
| Ctrl vs. Cu(II) | - | P <0.001*** |  |
| **Figure 7e** – Cell viability | Ordinary 1-way ANOVA | F = 10.4; P <0.001*** |  |
| *PAC2* | 95% confidence interval |  |  |
|  | Tukey's multiple comparison test |  |  |
| Ctrl vs. siYB-1 | - | P <0.001*** | 3 |
| Ctrl vs. Ctrl hydro. per. | - | P = 0.04* |  |
| Ctrl vs. siYB-1 hydro. per. | - | P = 0.002** |  |
| siYB-1 vs. Ctrl hydro. per. | - | P = 0.12 ns |  |
| siYB-1 vs. siYB-1 hydro. per. | - | P = 0.79 ns |  |
| Ctrl hydro. per. vs. siYB-1 hydro. per. | - | P = 0.49 ns |  |
| **Figure 8b** – Cell viability | Ordinary 1-way ANOVA | F = 0.5935; P = 0.70 ns | 6 |
| *Left graph (PAC2)* | 95% confidence interval |  |  |
|  | Tukey's multiple comparison test |  |  |
| Ctrl vs 100 µM hydro. per. | - | P >0.99 ns |  |
| Ctrl vs 200 µM hydro. per. | - | P = 0.94 ns |  |
| Ctrl vs 300 µM hydro. per. | - | P = 0.74 ns |  |
| Ctrl vs 600 µM hydro. per. | - | P = 0.79 ns |  |
| Ctrl vs 900 µM hydro. per. | - | P = 0.97 ns |  |
| **Figure 8b** – Cell viability | Ordinary 1-way ANOVA | F = 6.824; P <0.001*** | 4 |
| *Right graph (HDF)* | 95% confidence interval |  |  |
|  | Tukey's multiple comparison test |  |  |
| Ctrl vs 100 µM hydro. per. | - | P = 0.04* |  |
| Ctrl vs 200 µM hydro. per. | - | P = 0.01* |  |
| Ctrl vs 300 µM hydro. per. | - | P = 0.009** |  |
| Ctrl vs 600 µM hydro. per. | - | P = 0.002** |  |
| Ctrl vs 900 µM hydro. per. | - | P <0.001*** |  |
| **Figure S2c** – Aggregate size | Ordinary 1-way ANOVA | F = 52.54; P <0.001*** | 3 |
|  | 95% confidence interval |  |  |
| 26°C vs. 37°C | Dunnett's multiple comparison test | P >0.99 ns |  |
| 26°C vs. 40°C | - | P >0.99 ns |  |
| 26°C vs. 42°C | - | P = 0.50 ns |  |
| 26°C vs. 45°C | - | P <0.001*** |  |
| **Figure S5b** – RT-qPCR (*zf c-fos*) | Ordinary 1-way ANOVA | F = 13.24; P <0.001*** | 3 |
|  | 95% confidence interval |  |  |
| 0 vs. 30' | Dunnett's multiple comparison test | P = 0.01* |  |
| 0 vs. 1h | - | P = 0.02* |  |
| 0 vs. 3h | - | P = 0.46 ns |  |
| 0 vs. 6h | - | P = 0.42 ns |  |
| 0 vs. 9h | - | P = 0.33 ns |  |
| **Figure S5b** – RT-qPCR (*zf jun-B*) | Ordinary 1-way ANOVA | F = 93.63; P <0.001*** | 3 |
|  | 95% confidence interval |  |  |
| 0 vs. 30' | Dunnett's multiple comparison test | P <0.001*** |  |
| 0 vs. 1h | - | P <0.001*** |  |
| 0 vs. 3h | - | P = 0.54 ns |  |
| 0 vs. 6h | - | P = 0.91 ns |  |
| 0 vs. 9h | - | P >0.99 ns |  |
| **Figure S5b** – RT-qPCR (*zf jun-D*) | Ordinary 1-way ANOVA | F = 114.9; P <0.001*** | 3 |
|  | 95% confidence interval |  |  |
| 0 vs. 30' | Dunnett's multiple comparison test | P <0.001*** |  |
| 0 vs. 1h | - | P <0.001*** |  |
| 0 vs. 3h | - | P = 0.01* |  |
| 0 vs. 6h | - | P = 0.02* |  |
| 0 vs. 9h | - | P = 0.04* |  |
| **Figure S5b** – RT-qPCR (*zf cry1a*) | Ordinary 1-way ANOVA | F = 90.13; P <0.001*** | 3 |
|  | 95% confidence interval |  |  |
| 0 vs. 30' | Dunnett's multiple comparison test | P = 0.99 ns |  |
| 0 vs. 1h | - | P <0.001*** |  |
| 0 vs. 3h | - | P <0.001*** |  |
| 0 vs. 6h | - | P <0.001*** |  |
| **Figure S5b** – RT-qPCR (*zf cry5)* | Ordinary 1-way ANOVA | F = 16.53; P <0.001*** | 3 |
|  | 95% confidence interval |  |  |
| 0 vs. 30' | Dunnett's multiple comparison test | P = 0.85 ns |  |
| 0 vs. 1h | - | P = 0.002** |  |
| 0 vs. 3h | - | P = 0.001** |  |
| 0 vs. 6h | - | P <0.001*** |  |

**Table S2: RT-qPCR assay PCR primers**

| *zf β -actin* | F: GCCTGACGGACAGGTCAT | R: ACCGCAAGATTCCATACCC |
| --- | --- | --- |
| *zf yb-1* | F: TACCCACCATACTTCGTGCG | R: GCGGTAGTTGAAGTTGCGAC |
| *hyYb-1* | F: CGCAGTGTAGGAGATGGAGAG | R: GAACACCACCAGGACCTGTAA |
| zf *hsp70* | F: AGAGCATTACCCTGATGAAGC | R: TAAGTGGTGAAGGTCTGGGTCT |
| *zfcry1a* | F: TCCGCTGTGTGTACATCCTC | R: CAAACACTGCAGCAAAAACC |
| *Zfcry5* | F: ATGAGCCATAACACCATTCA | R TTATCTCTTTGCCTTCTTCTG: |
| *zf c-fos* | F: GCTCCATCTCAGTCCCAGAG | R: AGAGTGGGCTCCAGATCAGA |
| *zf junB* | F: GCTTCGTCAAGGCTCTGGAT | R: GTATGTGGGACGGCAGGTAG |
| *zf junD* | F: TTACACGAACTTGAGCGCCT | R: CGGTTACGCAGCTTCTTCCT |
| *zf gapdh* | F: CGACCACTTTGTCAAGCTCA | R: TTCCTCTTGTGCTCTTGCTG |

**Supplementary figures**

**
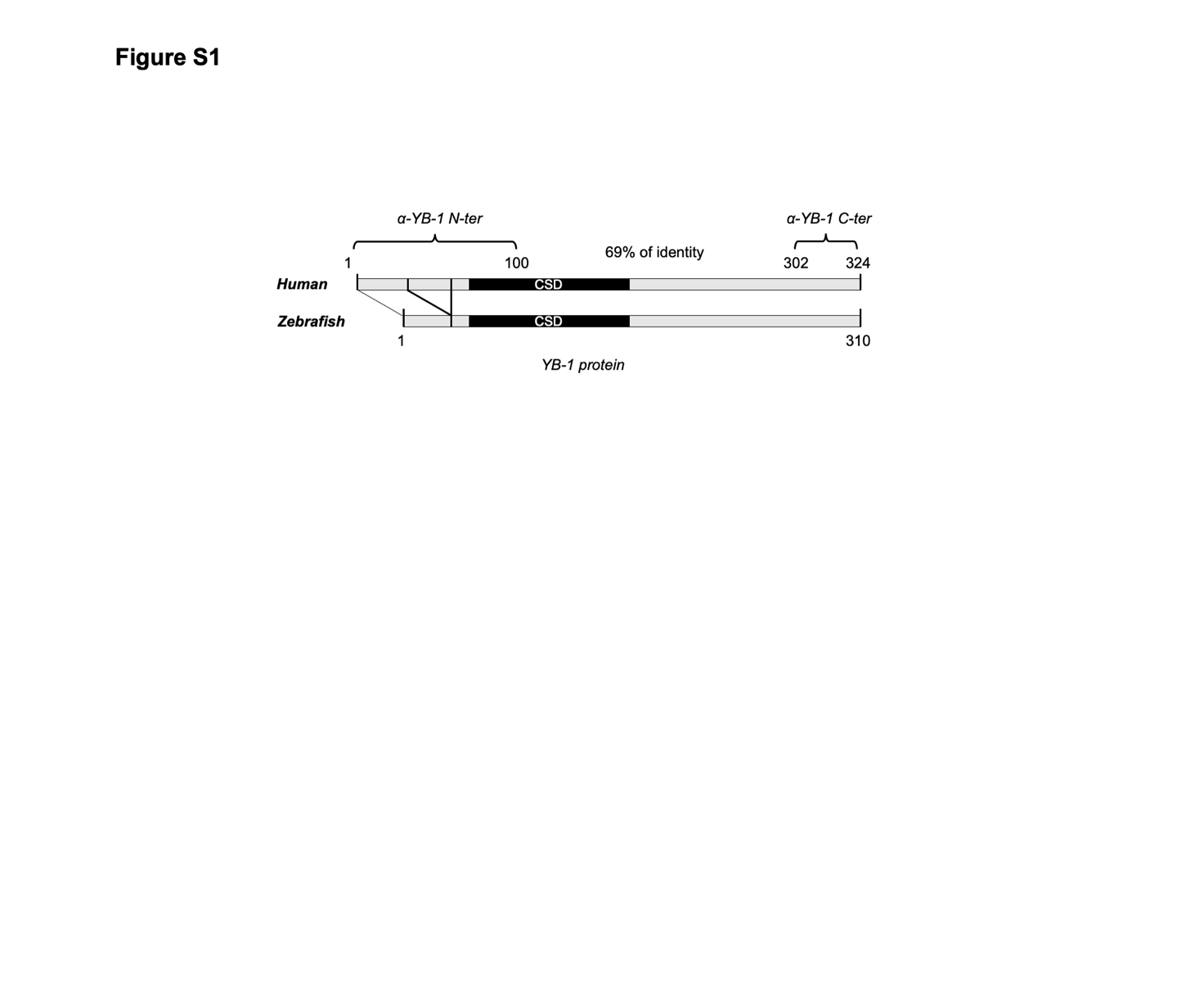
**

**Figure S1. Schematic representation of human and zebrafish YB-1 protein.**

Schematic representation of the human and zebrafish YB-1 proteins. Cold Shock Domains (black bars, CSD) and the portions recognized by the two antibodies, α-YB-1 N-ter and α-YB-1 C-ter are indicated.


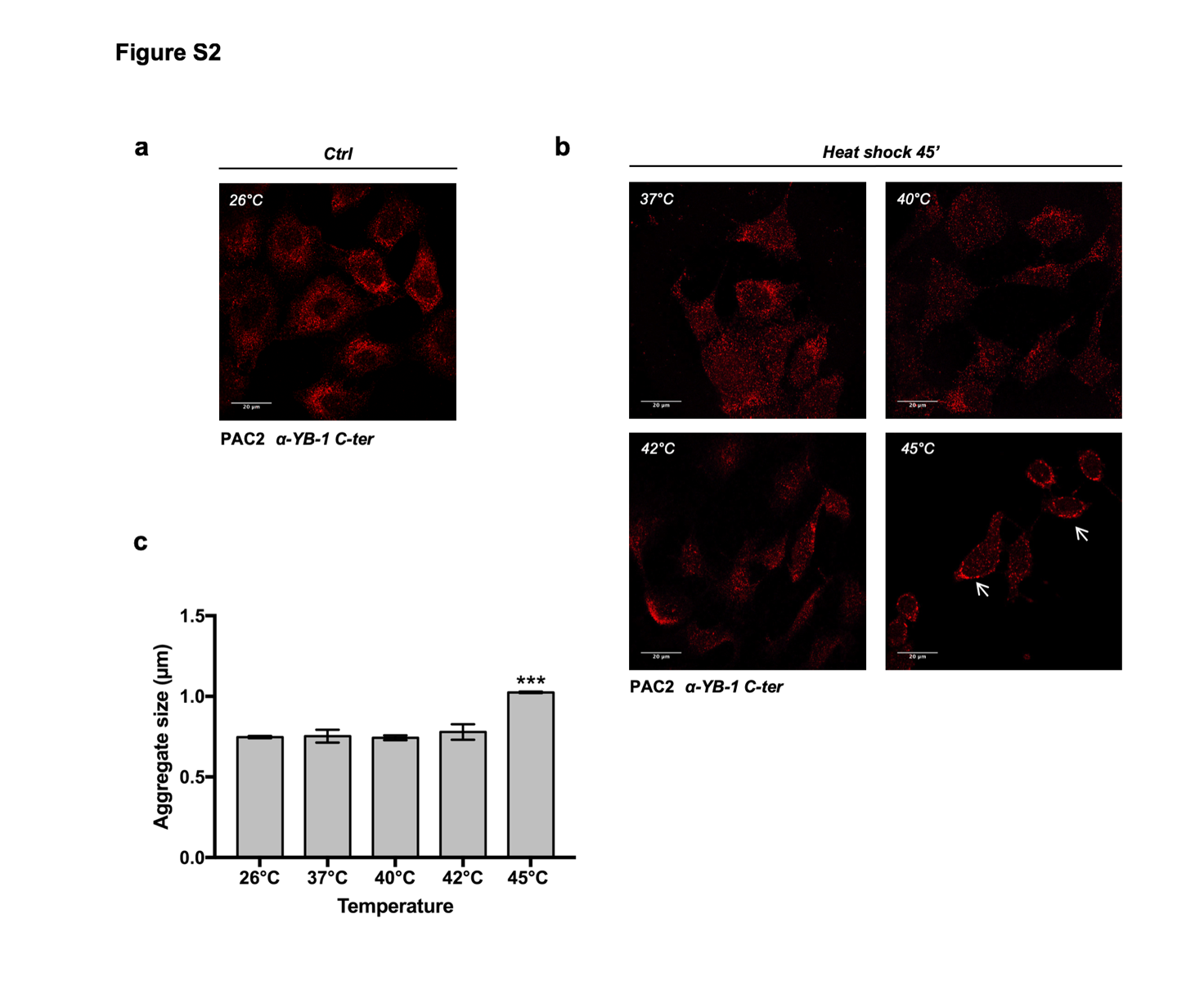


**Figure S2.** ***Bona fide* YB-1 SGs only form at 45°C.** **(a,b)** confocal immunofluorescence analysis of YB-1 (red) in PAC2 cells **(a)** at 26°C (Ctrl) and **(b)** after 45 minutes of heat shock at the indicated temperatures. YB-1 positive aggregates are indicated by white arrows; **c)** quantification of the dimensions of *bona fide* YB-1 SGs in PAC2 cells. Statistical analysis was performed using 1-way ANOVA followed Dunnett’s multiple comparisons test. Levels of significance are indicated (***<p 0.001) (see also Table S1).


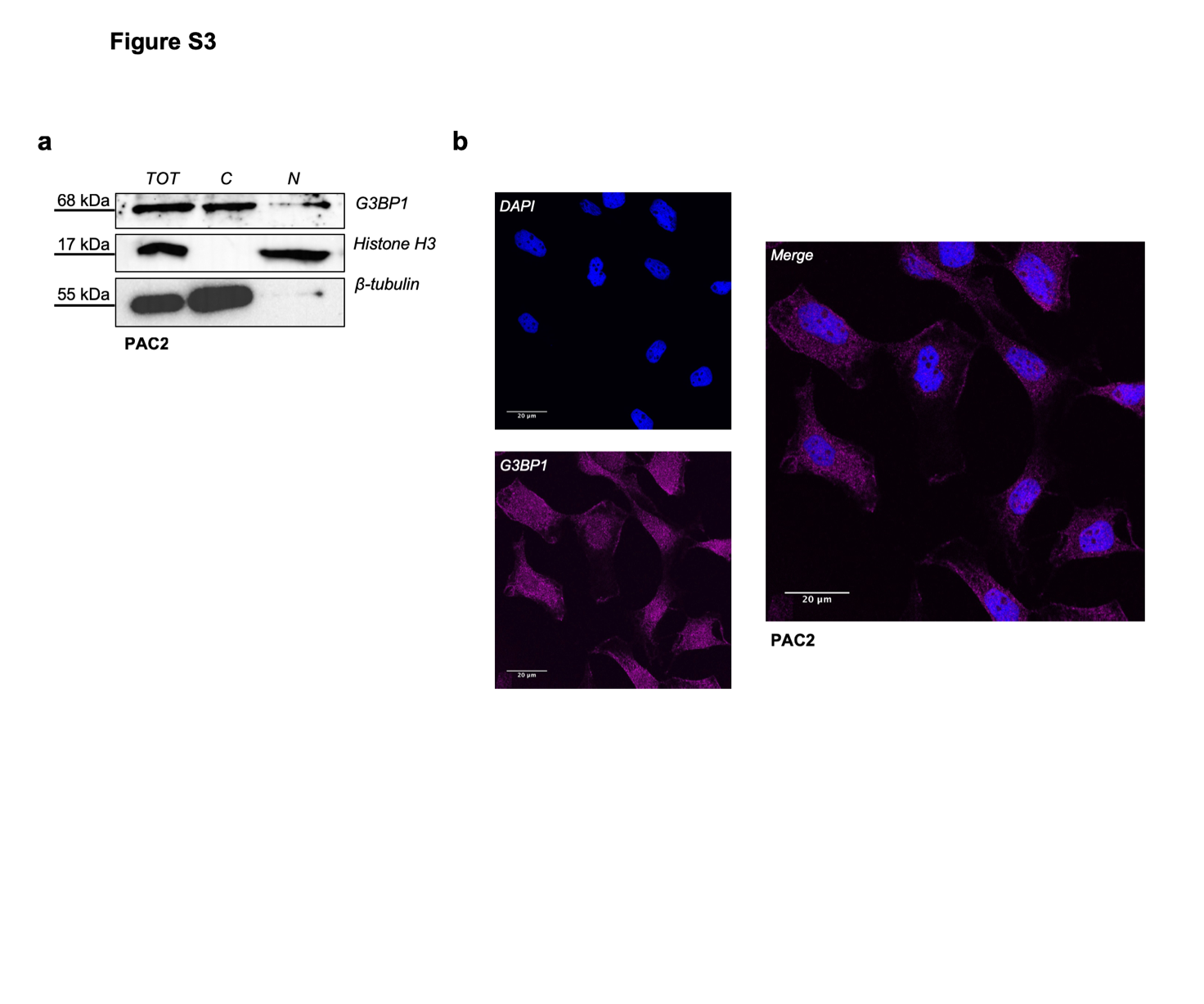


**Figure S3. Immunoreactivity of hG3BP1 antibody in zebrafish PAC2 cells. a)** western blot analysis using total extract and nuclear-cytoplasmic fractions of PAC2 cells incubated with human *α-*G3BP1 antibody. Histone H3 and ß-tubulin were used as loading controls for nucleus and cytoplasm, respectively. Each panel is assembled from cropped western blotting images (see Supplementary material file for the original images); **b)** confocal immunofluorescence analysis of G3BP1 (magenta) in PAC2 cells. Nuclei were stained with DAPI (blue) and Merge pictures are represented in the right part of the panel.


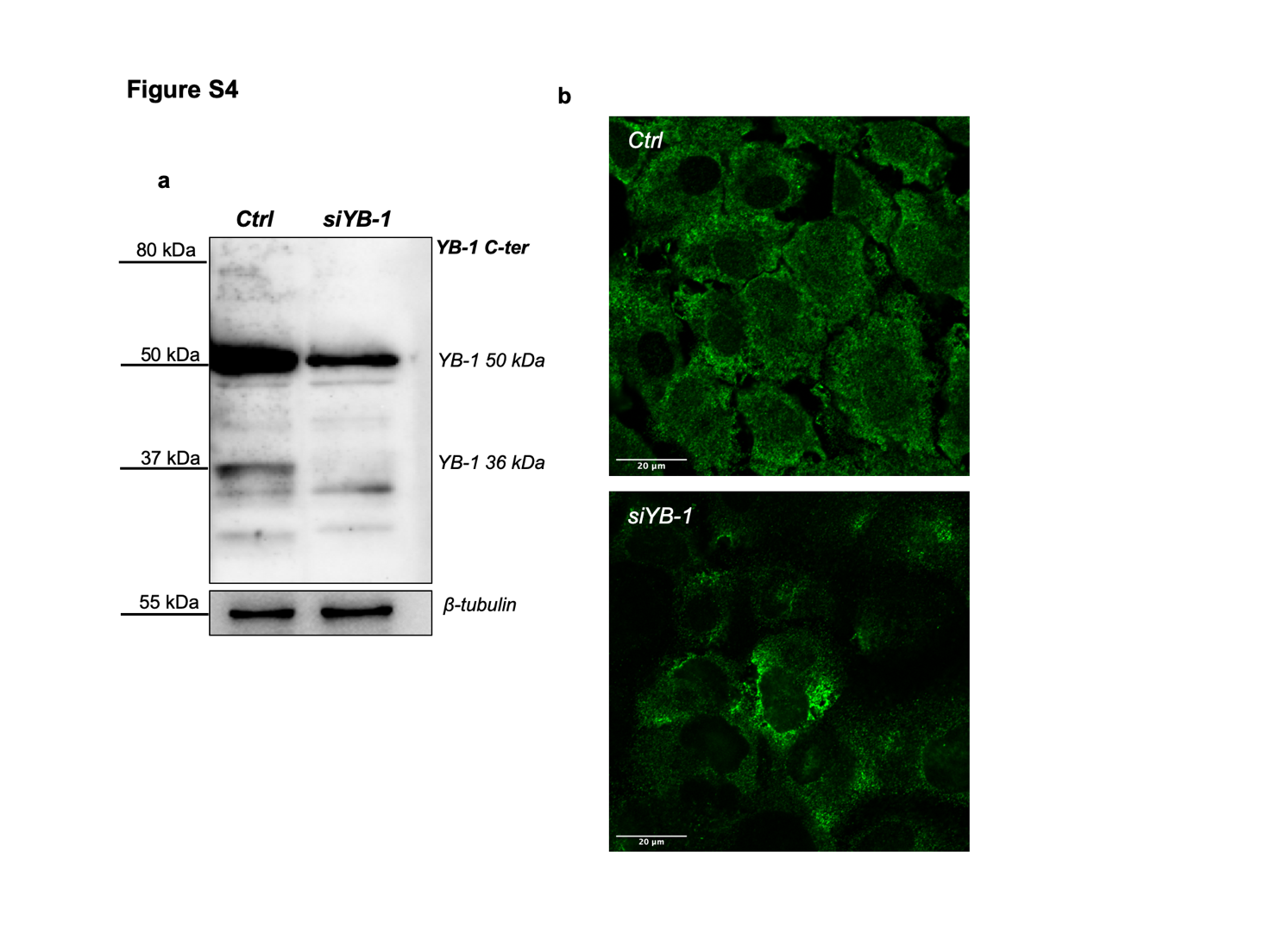


**Figure S4. Silencing of YB-1 in zebrafish PAC2 cells. a)** Western blot analysis of total protein extracts from control (*Ctrl*) and YB-1 silenced PAC2 cells (*siYB-1*). *ß-*tubulin was used as a loading control. Quantification of the YB-1 bands was performed using Image J software; the level of the 50 kDa YB-1 immunoreactive band in silenced cells was approximately 50 % of that in the control sample. The 36 kDa, as well as the high molecular weight YB-1 bands were undetectable in the siYB-1-treated sample. Each panel is assembled from cropped western blotting images (see Supplementary material file for the original images). **b)** Immunofluorescence assay for YB-1 in control (*Ctrl*) and YB-1 silenced PAC2 cells (*siYB-1*). The fluorescence signal in YB-1 silenced cells was 55% compared to the signal observed in cells transfected with the siRNA negative control (Riboxx) (see material and methods section for details). The quantification of the signals was performed using Image J.


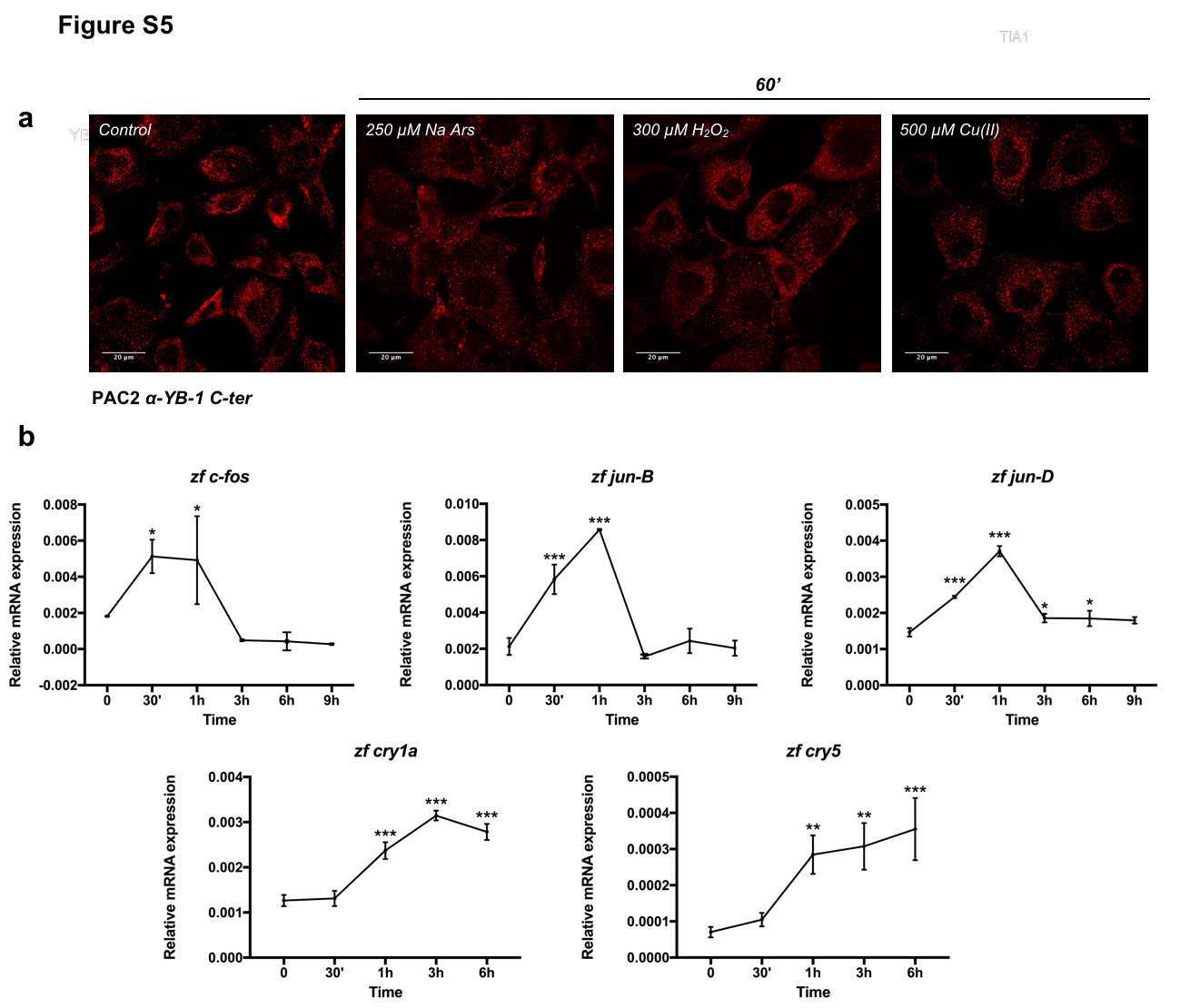


**Figure S5. PAC2 cells do not form SGs** **under oxidative stress.**  **a)** confocal immunofluorescence for YB-1 (red) in PAC2 cells treated with 250 µM Na Ars, 300 µM H_2_O_2_ and 500 µM Cu(II) for 60 minutes; **b)** RT-qPCR analysis of zebrafish *zf c-fos*, *zf jun-B*, *zf jun-D*, *zf cry1a* and *zf cry5* mRNA expression in PAC2 cells. Samples were taken at different time points after treatment with 300 µM H_2_O_2_. Mean mRNA relative expression (n=3) *±* SD is plotted on the y-axes, whereas time is plotted on the x-axes. Statistical analysis was performed using 1-way ANOVA followed by Dunnett’s multiple comparisons test. Levels of significance between the expression at each experimental timepoint and time 0 are indicated (*** p<0.001, **p<0.01, *p<0.05) (see also Table S1 for statistical analysis).


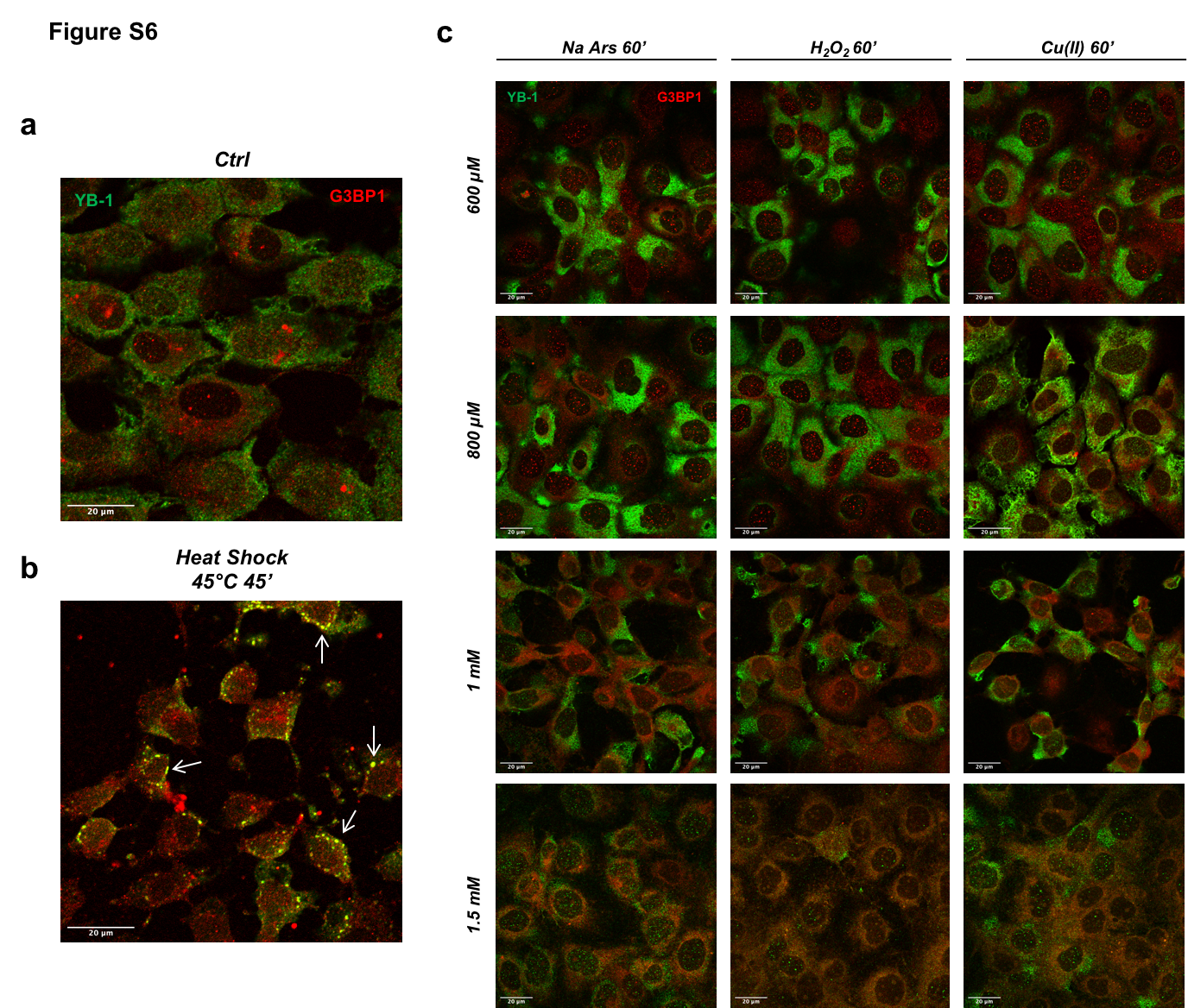


**Figure S6. Zebrafish cells do not form SGs** **even at higher oxidative stressor concentrations.**

**a-c)** Confocal immunofluorescence analysis of YB-1 (green) and G3BP1 (red) in PAC2 cells **a)** untreated PAC2 cells *(Ctrl),* **b)** PAC2 cells subjected to heat shock at 45°C for 45´. YB-1 and G3BP1 colocalization (yellow signal) in SG perinuclear granules is indicated by white arrows. **c)** PAC2 cells treated with Na Ars, H_2_O_2_ and Cu (II) at the concentrations indicated where no SG aggregates are visible.


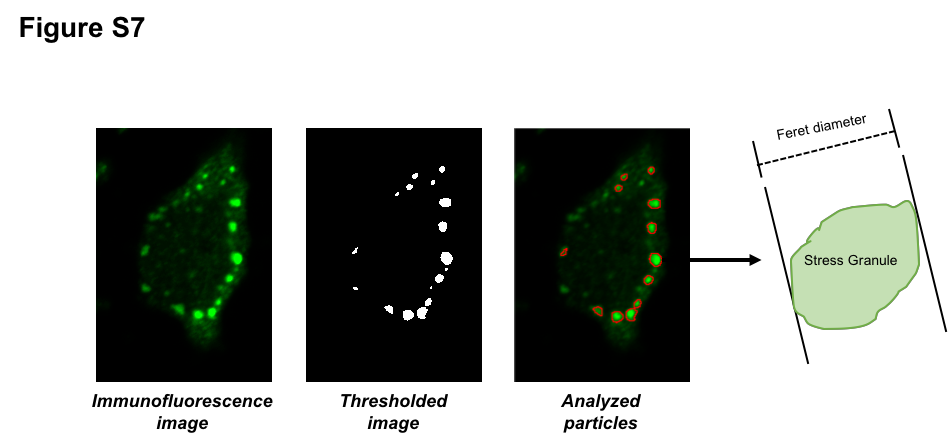


**Figure S7. SGs size measuring methods.** Representation of how the size of aggregates/stress granules was measured in confocal immunofluorescence images using Fiji (ImageJ) software to calculate the Feret’s statistical diameter. Feret‘s statistical diameter is the perpendicular distance between parallel tangents touching opposite sides of the profile of elliptical/circular shaped particles. This parameter is a reliable indicator of aggregate shape and dimensions. Analysis was performed on threshold images. Particles with areas outside the range of 0.2-2.0 µm and with size circularity values outside 1.00 were ignored.
