# Supplementary material for: YB-1 recruitment to stress granules in zebrafish cells reveals a differential adaptive response to stress
Source: Sci Rep. 2019 Jun 21;9:9059. doi: 10.1038/s41598-019-45468-6 (PMC6588705; doi:10.1038/s41598-019-45468-6)

**YB-1 recruitment to stress granules in zebrafish cells reveals a differential adaptive response to stress**

Andrea Maria Guarino^1^, Giuseppe Di Mauro^2,3^, Gennaro Ruggiero^2^, Nathalie Geyer^2^, Antonella Delicato^1^, Nicholas S. Foulkes^2^, Daniela Vallone^2*^ and Viola Calabrò^1*^.

.

^1^University of Naples Federico II, Department of Biology, Monte Sant’Angelo Campus, Via Cinthia 4, Naples, 80126, Italy.

^2^ Institute of Toxicology and Genetics, Karlsruhe Institute of Technology, Hermann-von-Helmholtz-Platz 1, 76344, Eggenstein-Leopoldshafen, Germany.

^3^ University of Ferrara, Department of Life Sciences and Biotechnology, Via Borsari 46, 44121, Ferrara, Italy.

*Co-senior and co-corresponding authors

Viola Calabrò, phone +39 081 679069 email: [vcalabro@unina.it](mailto:vcalabro@unina.it)

Daniela Vallone, phone +49 721 60828728 email: daniela.vallone@kit.edu

**Original western blotting data**

**Original western blotting data presented in Figure 1b (four panels).**
Margins of the original immunoblots are highlighted in black. Cropped areas of immunoblots used in the manuscript are indicated by red dash boxes. Antibodies used and molecular weights are indicated in each panel. For YB-1 protein, two major molecular forms (50 and 37 kDa) are indicated.

**
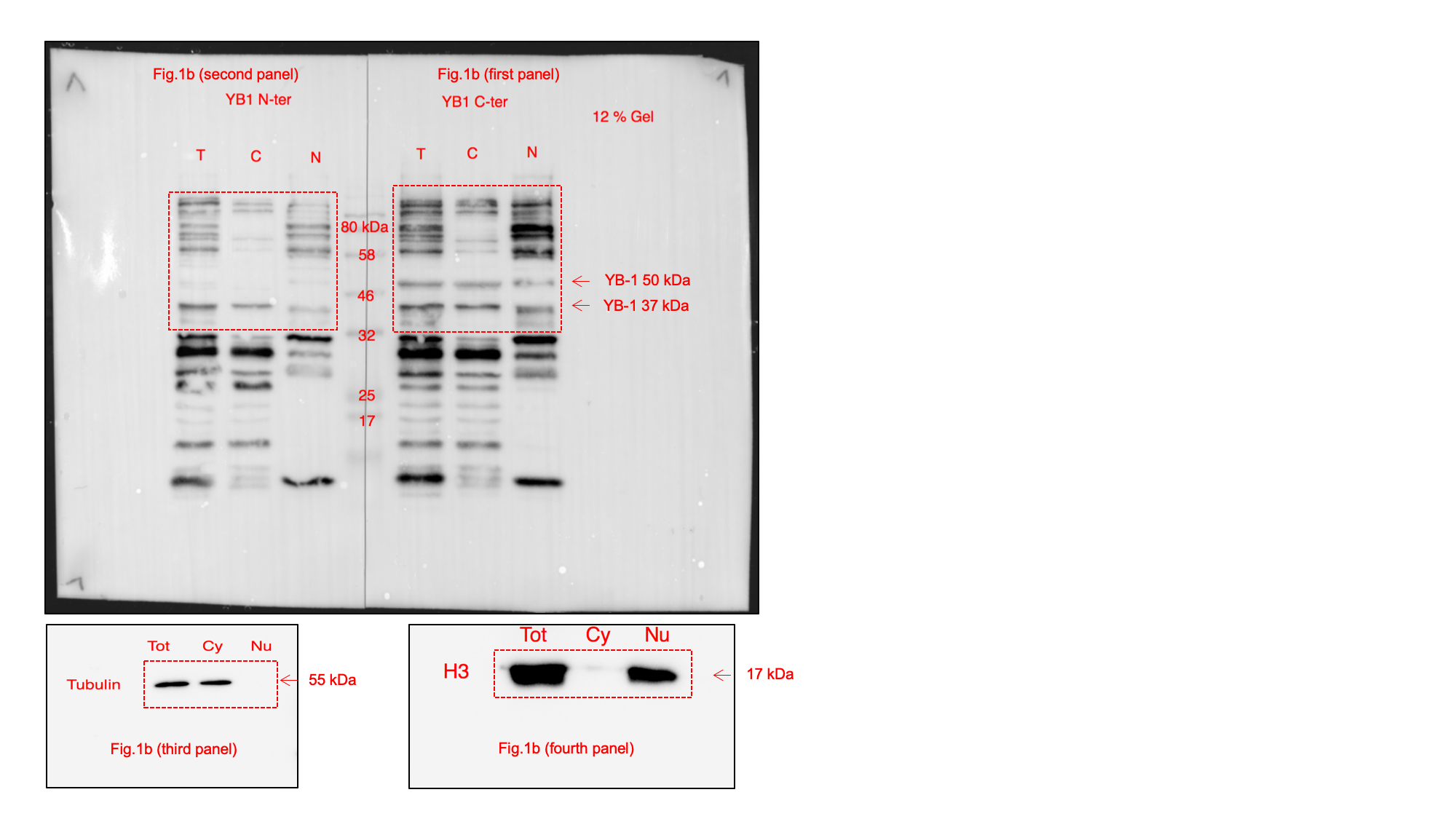
**

**Original western blotting data presented in Figure S3a (upper, middle and lower panels).**Cropped areas of immunoblots used in the manuscript are indicated by red dash boxes. TOT, C and N indicate, total protein extract, cytoplasmic protein extract and nuclear protein extract, respectively. Antibodies used and molecular weights are indicated in each panel.


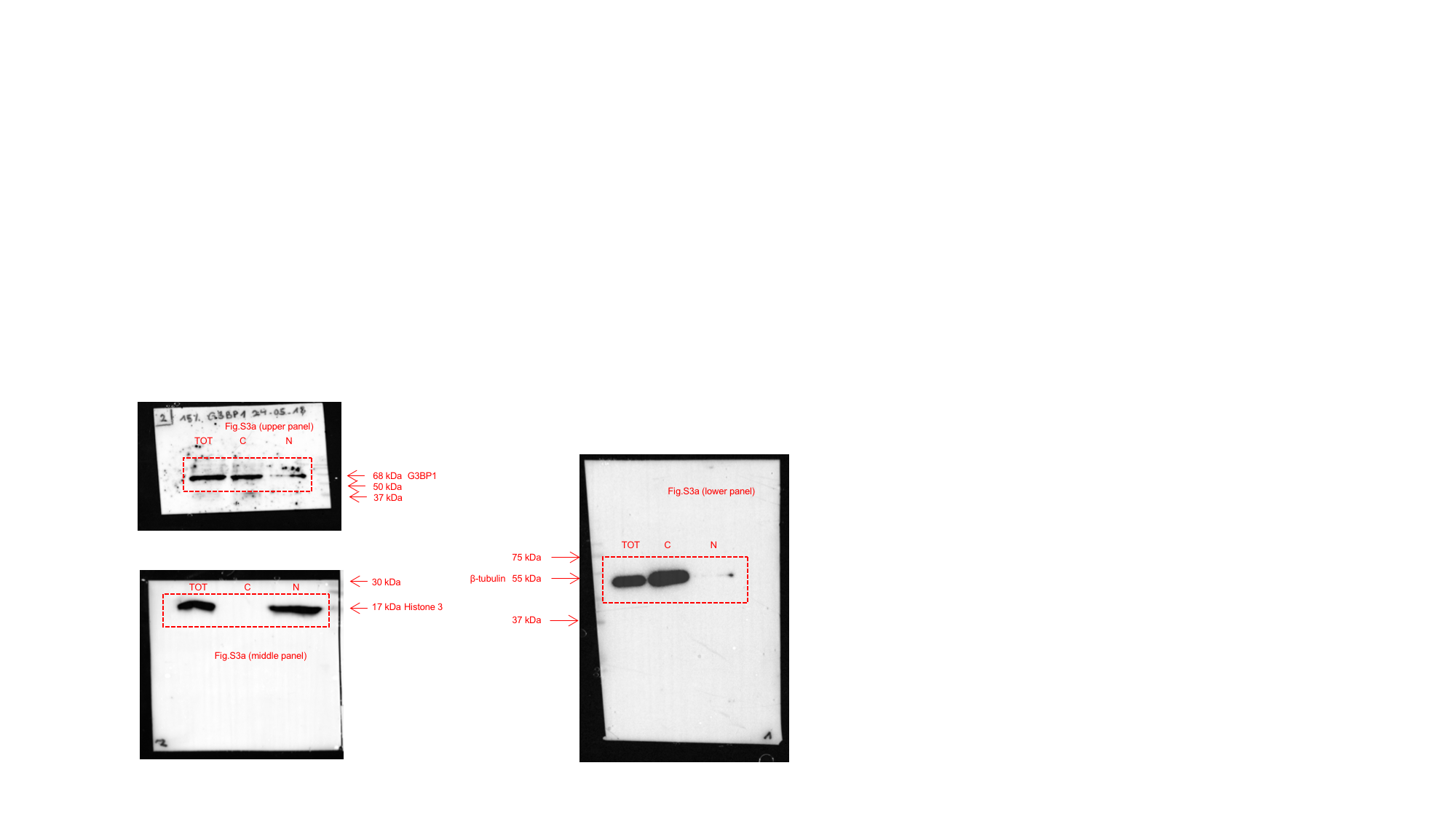


**Original western blotting data presented in Figure S4a.**
Margins of the original immunoblot are highlighted in black.
Cropped areas of immunoblots used in the manuscript are indicated by red dash boxes. Antibodies used and molecular weights are indicated in each panel.
For YB-1 protein, two major molecular forms (50 and 37 kDa) are indicated by arrows.


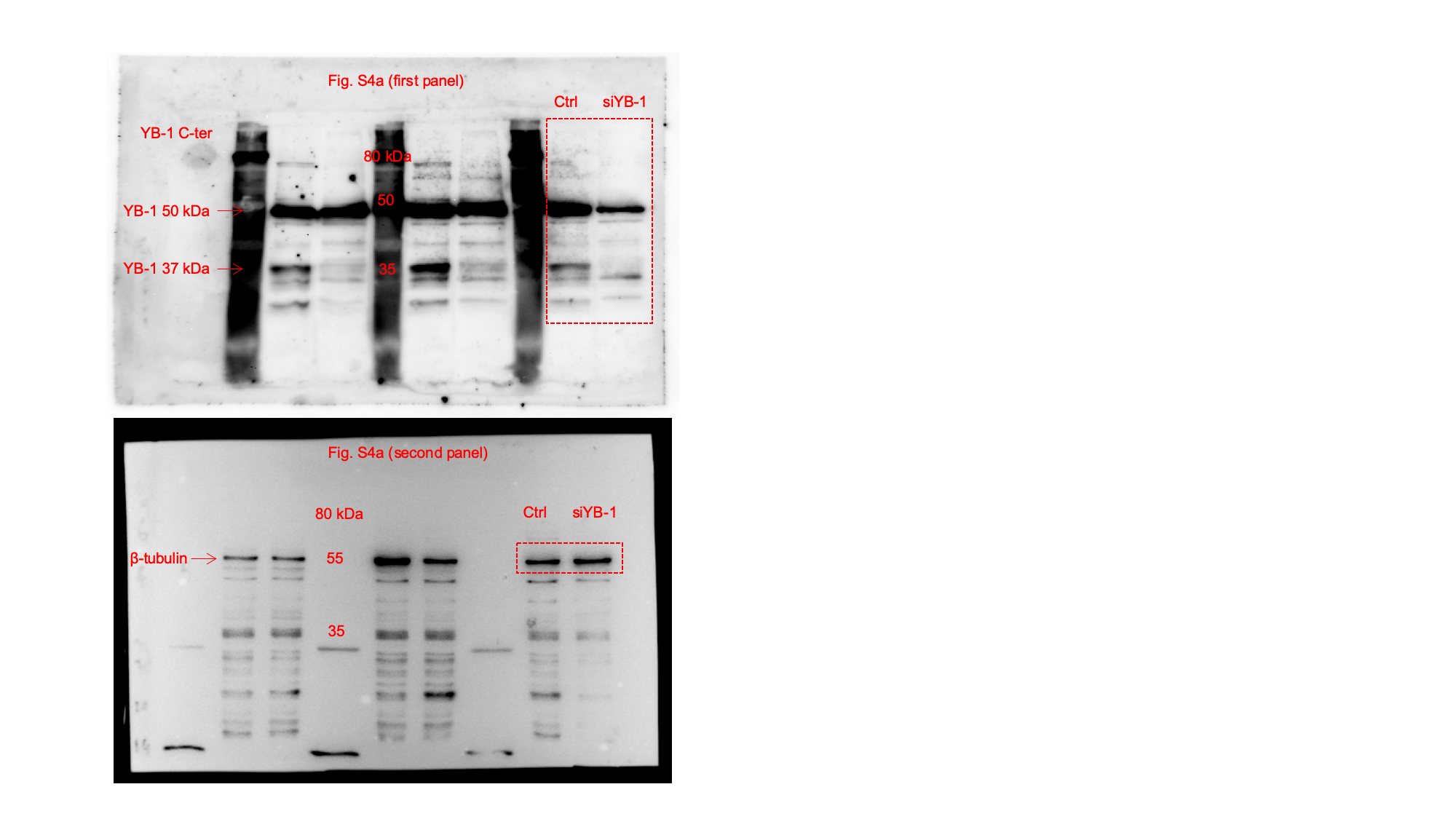

Supplement: Supplementary file 2 — Original Western-blot data [file 41598_2019_45468_MOESM2_ESM.docx]
